# Comparison of dexmedetomidine and dexamethasone as adjuvants to the ultrasound-guided interscalene nerve block in arthroscopic shoulder surgery: a systematic review and Bayesian network meta-analysis of randomized controlled trials

**DOI:** 10.3389/fmed.2023.1159216

**Published:** 2023-06-16

**Authors:** Xiu-Min Wei, Zheng Liu, Lian-Chao Lv, Guang-Han Wu, Peng-Yu Sun, Chang-Ping Gu, Peng-Cai Shi

**Affiliations:** ^1^Department of Anesthesiology, The First Affiliated Hospital of Shandong First Medical University & Shandong Provincial Qianfoshan Hospital, Shandong Institute of Anesthesia and Respiratory Critical Medicine, Jinan, Shandong, China; ^2^School of Anesthesiology, Weifang Medical University, Weifang, Shandong, China; ^3^Department of Anesthesiology, Shandong Provincial Hospital Affiliated to Shandong First Medical University, Jinan, Shandong, China

**Keywords:** arthroscopic shoulder surgery, interscalene nerve block, adjuvants, dexamethasone, Bayesian network meta-analysis

## Abstract

**Introduction:**

Interscalene block (ISB) is widely regarded as the gold standard treatment for acute pain following arthroscopic shoulder surgery. However, a single injection of a local anesthetic for ISB may not offer sufficient analgesia. Various adjuvants have been demonstrated to prolong the analgesic duration of the block. Hence, this study aimed to assess the relative efficacy of dexamethasone and dexmedetomidine as adjuncts to prolong the analgesic duration for a single- shot ISB.

**Methods:**

The efficacy of adjuvants was compared using a network meta-analysis. The methodological quality of the included studies was evaluated using the Cochrane bias risk assessment tool. A comprehensive search of the PubMed, Cochrane, Web of Science, and Embase databases was conducted with a search deadline of March 1, 2023. Various adjuvant prevention randomized controlled trials have been conducted in patients undergoing interscalene brachial plexus block for shoulder arthroscopic surgery.

**Results:**

Twenty-five studies enrolling a total of 2,194 patients reported duration of analgesia. Combined dexmedetomidine and dexamethasone (MD = 22.13, 95% CI 16.67, 27.58), dexamethasone administered perineurally (MD = 9.94, 95% CI 7.71, 12.17), high-dose intravenous dexamethasone (MD = 7.47, 95% CI 4.41, 10.53), dexmedetomidine administered perineurally (MD = 6.82, 95% CI 3.43, 10.20), and low-dose intravenous dexamethasone (MD = 6.72, 95% CI 3.74, 9.70) provided significantly longer analgesic effects compared with the control group.

**Discussion:**

The combination of intravenous dexamethasone and dexmedetomidine provided the greatest effect in terms of prolonged analgesia, reduced opioid doses, and lower pain scores. Furthermore, peripheral dexamethasone in prolonging the analgesic duration and lowering opioid usage was better than the other adjuvants when used a single medication. All therapies significantly prolonged the analgesic duration and reduced the opioid dose of a single-shot ISB in shoulder arthroscopy compared with the placebo.

## 1. Introduction

Interscalene block (ISB) is widely regarded as the gold standard for the treatment of acute pain following arthroscopic shoulder surgery as it provides great analgesia in the early postoperative period while reducing opioid consumption and adverse effects (e.g., respiratory depression, nausea, and vomiting) (1, 2). However, a single injection of local anesthetic for ISB may not offer sufficient analgesia if used for longer than 14 h. The short duration of analgesia and analgesic effect of a single shot of ISB restrict its use (3). Continuous infusion analgesia using a patient-controlled interscalene catheter might prolong the duration of analgesia; unfortunately, it cannot circumvent the inherent practical problems and complications of plexus catheter infusion maintenance (4).

Various adjuvants have been demonstrated to prolong the analgesic duration of the block, including epinephrine, clonidine, dexmedetomidine, and intravenous and perineural injection of dexamethasone (3, 5). As a highly selective α_2_ adrenergic receptor agonist, dexmedetomidine is anticipated to have a longer analgesic duration than other adjuvants, without neurotoxicity (6). Dexamethasone, a potent glucocorticoid, is effective at both low (4 mg) and high (8 mg) concentrations. Several animal experiments have proven that these adjuvants prolong the impact of a nerve block, and clinical trials have also verified the beneficial effects on peripheral nerve and brachial plexus block (7). However, different doses and modes of administration of adjuvant therapies affect analgesic duration extension, and a quantitative evaluation of their efficacy is still required.

The objective of this network meta-analysis was to determine the relative efficacy of dexamethasone and dexmedetomidine as adjuncts to prolong the analgesic duration of a single-shot ISB.

## 2. Method

This systematic review and meta-analysis followed the Preferred Reporting Items for Systematic Reviews and Meta-Analyses (PRISMA) guideline and the Cochrane Handbook for the Systematic Review of Interventions (8, 9). The research reviewed the existing data; thus, neither ethical approval nor patient agreement was necessary.

### 2.1. Search strategy

Two authors independently designed and conducted a systematic literature search to identify the parallel group and cross-over randomized controlled trials (RCTs) on PubMed, Embase, Web of Science, the Cochrane Central Register of Controlled Trials, the China National Knowledge Infrastructure database, the Chinese Scientific Journal database, and the Wan Fang Database with a search deadline of March 1, 2023. Without any restrictions on the publication year, region, or language, our search method included Medical Topic Headings (MeSH), Emtree phrases, subject headings, and free-text terms, mainly including the following terms: “arthroscopic shoulder surgery,” “dexamethasone,” “dexmedetomidine,” and “adjuncts.” We conducted further analysis to determine whether the material was provided in a non-English language.

In addition, we searched the bibliography lists of relevant previous studies to perform a battery of recursive searches and manual retrieval for potential studies, where only abstracts meeting our eligibility criteria were presented. EndNote X9 was used to manage all the above screening records (Thomson ISI Research Soft, Philadelphia, PA, USA). A comprehensive list of search phrases for each database is available in the “Search Strategies” supplement.

### 2.2. Eligibility criteria and data abstraction

The inclusion and exclusion criteria were prioritized according to the PICO criteria. RCTs published in peer-reviewed scientific journals compared the efficacy of adjuvants for ISB to control postoperative pain in arthroscopic shoulder surgery. The PICO criteria were classified as follows: Participants: Patients scheduled for elective shoulder arthroscopic surgery were enrolled in this network meta-analysis (NMA). Interventions: Intravenous or perineural injection was administered as adjuvants for ISB in patients undergoing shoulder arthroscopic surgery. Comparators: Interventions themselves or patients who received an ISB alone. Outcomes: The primary outcome was analgesia duration. The postoperative analgesic duration was defined as the time interval between ISB and the request for the first rescue analgesic, the time of the first-time shoulder pain was experienced, and the sensory block duration. The secondary outcomes were opioid consumption and pain score. Consumption of opioids is defined as the use of oral morphine equivalents, according to the general monograph for opioids in the Canadian Pharmacists' Association Compendium of Pharmaceuticals and Specialties. Opioid consumption was converted to morphine equivalents using standard conversions (10). The determination of pain is mainly based on the Visual Analog Scale (VAS) or Numeric Rating Score (NRS), which defines the presence and degree of pain. Different pain levels can be measured on a scale of 0–10, with 0 representing no pain and 10 representing the worst pain. Study design: This review included both parallel-group and crossover RCTs. The studies were divided into six groups: low-dose intravenous dexamethasone (4 mg) (low-dose DXM-IV), high-dose intravenous dexamethasone (8 mg) (high-dose DXM-IV), perineural dexamethasone (DXM-PN), perineural dexmedetomidine (DEX-PN), combined intravenous dexamethasone and dexmedetomidine (DEX-DXM), and control group. Two authors (X-MW and ZL) separately identified the relevant articles. Initial searches were conducted on both the titles and abstracts using the defined eligibility criteria. In this phase, duplicate articles were removed from the retrieved articles simultaneously.

All articles selected from the initial research were retrieved and assessed based on their full text. If no data were available for abstract-only research, they were disregarded. Disagreements were settled by discussions between reviewers and consultation with an independent expert referee (P-CS) to ensure that a consensus was reached on all items.

### 2.3. Outcome measurement and quality appraisal

According to a pre-tested, nine-item, standardized data extraction form, two independent authors extracted data from each article under the following headings: first author(s), year of publication, patient characteristics, sample size, duration of analgesia, pain scores, opioid consumption, and incidence of complications. The mean and standard deviation (SD) of the duration of analgesia, pain scores, and opioid consumption were extracted as continuous outcomes. If the duration of analgesia and VAS was expressed as median with interquartile range (IQR), it was transformed and expressed as mean ± SD before statistical analysis (8). We presumed that the width of the IQR was equal to 1.35 times the SD and that the median was equal to the mean. The formulas used to get the mean and standard deviation (SD) were all based on the recommendations provided in the Cochrane Handbook for Systematic Reviews (11).

Two independent authors (ZL and X-MW) evaluated and categorized the risk of bias (ROB) using the Cochrane Handbook version 5.1.0 tool in Review Manager (version 5.3) (8). For each trial, we categorized the risk as low, high, or unclear, according to the seven assessment items. For selection bias, we evaluated whether the studies clearly defined the random sequence generation and allocation concealment method. Regarding detection bias, we evaluated studies primarily based on whether the participants, personnel, and outcome evaluators were blinded. We classified patients as high-risk for attrition bias in studies in which essential data were missing, particularly primary outcome data. We assessed selection bias based on whether the research excluded secondary outcomes or provided inadequate data. Other biases were categorized based on a full-text search for evidence that may have contributed to potential inconsistencies among the included studies. In addition, the GRADE method was used to evaluate the quality of evidence for each connection (12).

### 2.4. Statistical analysis

A network plot was generated to simulate a fully connected network, as an overview of the available evidence for each adjuvant. A comparison-adjusted network funnel plot was used to visually assess publication bias. Both analyses were conducted using the Stata software (version 14.0; Stata Corp, College Station, TX, USA).

Transitivity is the key underlying assumption of the NMA and relates to the validity of making indirect comparisons and the homogeneous distribution of effect modifiers across the included studies. Before performing data analysis, the baseline characteristics of the participants were presented for each intervention group (13–15). We assigned a non-informative prior distribution to the parameters based on a Bayesian framework (16). The Markov chain Monte Carlo method was used to examine all the results, which established three distinct chains with a total number of 50,000 iterations (17–19). For continuous variables, we used the mean difference (MD) to pool the effect size and 95% confidence intervals (CIs) (20, 21). The proportion of the best ranking in all simulated activities was used to calculate the probability of which adjuvant intervention would be the best. For each treatment, the surface under the cumulative ranking curve (SUCRA) was used to estimate cumulative ranking (22). The SUCRA value is presented as a percentage, ranging from 0 to 100%. Higher SUCRA values indicate a better ranking of treatment effectiveness, whereas lower SUCRA values indicate a worse trend (21). By evaluating the trace “history” feature, both the tract plot and the Brooks-Gelman-Rubin diagnostic statistics were considered to ensure convergence. Sensitive analysis by omitting one study in each turn was performed. The above analyses were performed using STATA (ver. 14.2; StataCorp, Lakeway Drive College Station, TX 77845, USA) and OpenBUGS (ver. 3.2.3 rev 1012, Members of OpenBUGS Project Management Group) software. Details of the OpenBUGS code are presented in the “OpenBUGS code” supplement. The node-splitting method was used to assess model inconsistency, where the probability of significant inconsistency was indicated if node-splitting analysis-derived *P*-values were < 0.05 (8, 18, 23, 24). *I*^2^ statistic was tested for assessing substantial heterogeneity, of which the values 25, 50, 75% indicated mild, moderate and high heterogeneity respectively (9). The analysis was performed using “Gemtc” package (version 0.8–2) and “rjags” (version 4–6) in R language (X64 4.12 version).

## 3. Results

### 3.1. Baseline characteristics and quality of the included studies

**Figure 1:**
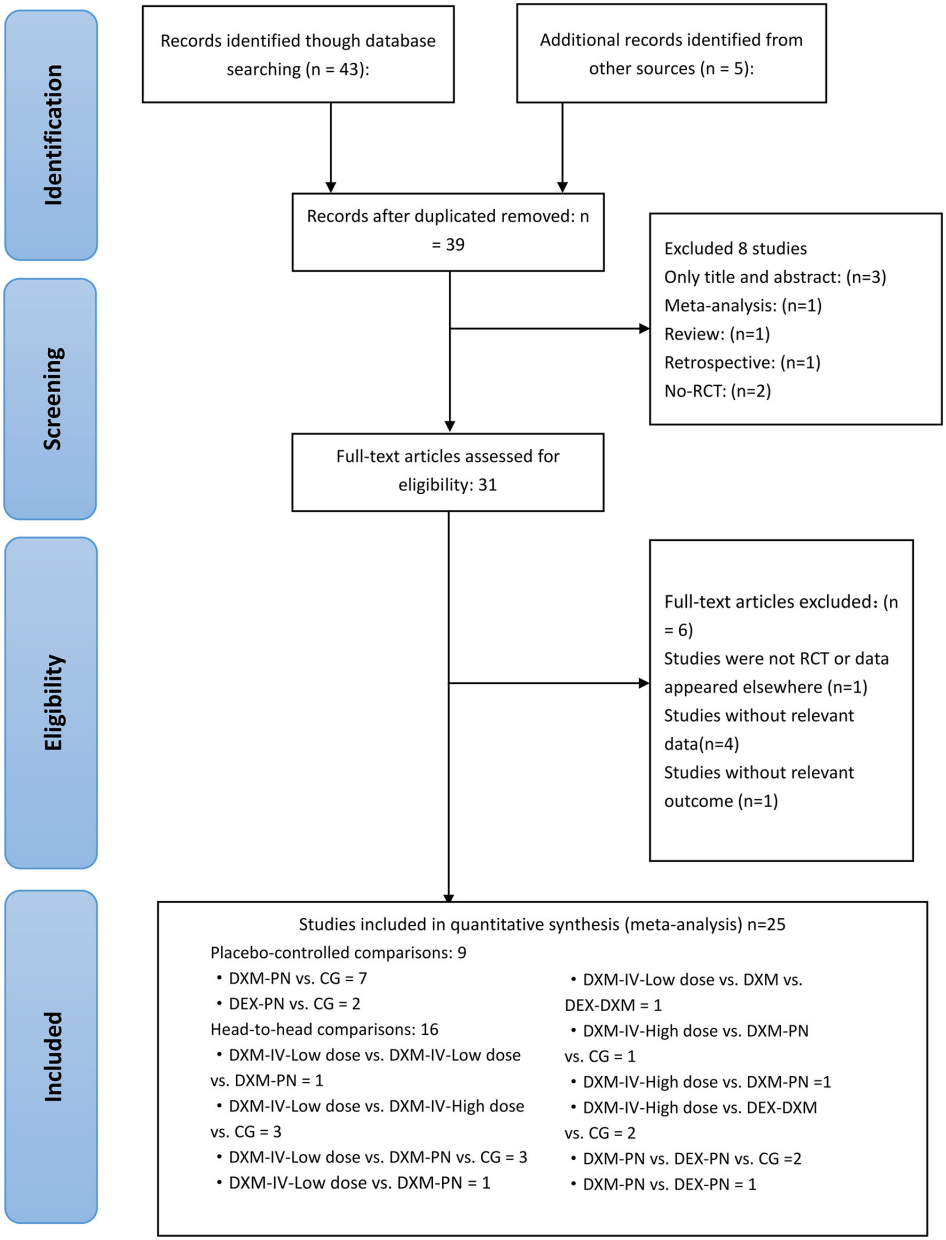
Literature review flowchart; RCT, Randomized controlled trial; CG, Control group. DXM, dexamethasone; DEX, dexmedetomidine; IV, intravenous; PN, perineural.

Initially, a total of 48 studies were identified by searching electronic databases and manually, of which 9 articles were removed due to duplication. Furthermore, 3 were excluded owing to irrelevant topics after screening based on the titles and abstracts. Following the full-text screening, 25 articles remained, and 23 articles were excluded for the following reasons: 4 did not report relevant data, 1 did not contain relevant outcomes, 1 was not an RCT, and 1 did not report relevant outcomes. Eventually, a total of 25 RCTs were deemed eligible for review and inclusion in this NMA, and a unanimous agreement was achieved on all articles among the reviewer authors. The literature search and study selection procedures are presented in Figure 1. EndNote X9 software (Clarivate Analytics, London, United Kingdom) was used to import and maintain all reference lists retrieved using a search engine. Table 1 summarizes the essential characteristics of the included studies.

**Table 1:** Characteristics of included studies.

**ID**	**Study**	**Year**	**Total**	**Age**	**Gender (M/F)**	**ASA**	**Primary anethesia**	**Main outcome**	**Ultrasound used**	**Amount and type of anesthetic agent**	**Intervention**	**Outcome**
1	Rodrigues et al. (25)	2021	197	50.3 ± 14.1 vs. 52.5 ± 13.87 vs. 49.4 ± 12.7	45/21 vs. 49/16 vs. 53/13	I-III	General anesthesia with a single-shot ISB	The primary outcome was duration of analgesia after ISB as measured by time from block administration to the first time shoulder pain was experienced after the surgery.	Y	30 ml 0.5% bupivacaine	dexmedetomidine 50 mcg PN, dexamethasone 4 mg I.V., dexmedetomidine 50 mcg + dexamethasone 4 mg I.V.	DOA/O
2	Woo et al. (26)	2021	70	62.2 ± 8.0 vs. 58.8 ±9.6	15/20 vs. 20/15	I-III	General anesthesia with a single-shot ISB	The time of the first analgesic request was recorded as the interval between ISB and the first analgesic administration.	Y	12 ml of 0.5% ropivacaine	dexamethasone 5 mg PN.	DOA/O/VAS
3	Holland et al. (7)	2017	209	53 ± 14 vs. 50 ± 15 vs. 51 ± 14	53/16 vs. 45/25 vs. 45/25	I-III	General anesthesia with a single-shot ISB	The primary outcome was defined as the duration of ISB analgesia, measured from the time of completion of the injection of the ISB solution to the time the patient first experienced shoulder pain after surgery.	Y	30 ml 0.5% bupivacaine	dexamethasone 4 mg or 8mg I.V. or 8 mg PN.	DOA/O
4	Chalifoux et al. (27)	2016	69	54.7 ± 7.4 vs. 54.7 ± 10.5 vs. 48.8 ± 12.4	11/11 vs. 12/11 vs. 17/7	not mention	General anesthesia with a single-shot	The first analgesic request occurred.	Y	20 ml 0.5% ropivacaine	dexamethasone 4 mg I.V., dexamethasone 10 mg I.V.	DOA/O/VAS
5	Kang et al. (1)	2019	66	46.3 ± 16.6 vs. 46.1 ± 17.0 vs. 47.4 ±13.5	14/8 vs. 15/7 vs. 13/9	I-II	General anesthesia with a single-shot ISB	The time to first rescue analgesic request.	Y	15 ml of 0.5% ropivacaine	dexamethasone 0.11 mg/kg I.V.; dexamethasone 0.11 mg/kg + dexmedetomidine 1.0 mcg/kg I.V.	DOA/O
6	Kataria et al. (28)	2019	60	30.13 ± 10.89 vs. 30.17 ± 11.69	25/5 vs. 24/6	not mention	General anesthesia with a single-shot ISB	Duration of analgesia (defined as time from set of adequate sensory block to the time of patient self-administering the first bolus of supplemental an algesic medication).	Y	20 ml 0.5% ropivacaine	dexmedetomidine 0.5 mcg/kg PN; dexamethasone 8 mg PN.	DOA/O
7	Chun et al. (29)	2016	99	53.0 ± 14.2 Vs. 50.8 ± 17.5	34/15 vs. 33/17	I-III	General anesthesia with a single-shot ISB	The time to the first analgesic request.	Y	12 ml of ropivacaine 5mg	Dexamethasone 5 mg I.V., dexamethasone 5 mg PN.	DOA/VAS
8	Jadon et al. (30)	2015	112	male:48.36 ± 13.82 female: 50.96 ± 10.10 vs. male:48.72 ± 12.75 female: 51.14 ± 10.83	25/25 vs. 22/28	I-II	General anesthesia with a single-shot ISB	Primary outcome measure was to evaluate the effect of mixing dexamethasone on duration of analgesia provided by ISB.	N	30 ml of 0.5% ropivacaine	Dexamethasone 8 mg PN.	DOA/VAS
9	Desmet et al. (31)	2013	144	51.1 ± 14.3 vs. 53.0 ±13.9 vs. 51.6 ± 14.0	21/25 vs. 21/28 vs. 23/26	not mention	General anesthesia with a single-shot ISB	The primary outcome was the duration of analgesia, defined as the time between performance of the block and the first analgesic request.	Y	30 ml of 0.5% ropivacaine	dexamethasone 10 mg I.V.; dexamethasone 10 mg PN.	DOA
10	Woo et al. (32)	2015	72	54.4 ± 17.8 vs. 47.3 ± 17.5	23/13 vs. 30/6	I-II	General anesthesia with a single-shot ISB	The primary endpoint was the time to the first analgesic request.	Y	12 ml of 0.5% ropivacaine	dexamethasone 5 mg PN.	DOA/VAS
11	McHardy et al. (33)	2019	179	51.6 ± 13.75 vs. 52.8 ± 13.5	67/25 vs. 69/21	I-III	General anesthesia with a single-shot ISB	The primary outcome was duration of sensory block defined as the time from the end of injection to the first sensation of pain at the surgical site.	Y	5 ml of 0.5% ropivacaine	dexamethasone 4 mg I.V. or PN.	DOA/O/VAS
12	Kawanishi et al. (4)	2014	34	56.7 ± 16.6 vs. 55.6 ± 12.8vs. 59.2 ± 15.3	8/4 vs. 9/3 vs. 7/3	I-II	General anesthesia with a single-shot ISB	The primary outcome was the duration of analgesia, defined as the time between performance of the block and the first request for analgesic.	Y	20 ml of 0.75% ropivacaine	dexamethasone 4 mg PN., dexamethasone 4 mg I.V.	DOA
13	Jung et al. (3)	2018	47	58.70 ± 11.19 vs. 58.67 ± 7.74	7/16 vs. 15/9	I-II	General anesthesia with a single-shot ISB	The primary outcome was the duration of analgesia.	Y	20 ml of 0.5% ropivacaine	dexmedetomidine 2 mcg/kg PN	DOA
14	Lin et al. (34)	2017	60	48.5 ± 8.1 vs. 50.8 ± 8.7 vs. 47.0 ± 9.0	10/10 vs. 11/9 vs. 12/8	I-II	General anesthesia with a single-shot ISB	Primary endpoint was the time to first postoperative analgesic required.	Y	30 ml of 0.5% ropivacaine	dexamethasone 0.05 mg/kg I.V.; dexamethasone 0.1 mg/kg I.V.	DOA
15	Margulis et al. (35)	2021	89	52 ± 3 vs. 54 ± 2.5 vs. 52 ± 4.5	19/9 vs. 12/18 vs. 19/12	I-II	General anesthesia with a single-shot ISB	The time to first postoperative opioid required.	Y	20 ml of 0.5% ropivacaine	dexamethasone 4 mg PN; dexmedetomidine 75 mcg PN	DOA/O/VAS
16	Vasconcelos et al. (36)	2020	71	47.2 ± 13 vs. 50.7 ± 11	46/54 vs. 59/41	I-II	General anesthesia with a single-shot ISB	The duration of the sensory block.	Y	30 ml of 0.5% levobupivacaine	dexamethasone 6 mg PN.	DOA/VAS
17	Morita et al. (2)	2020	54	62.1 ± 11.0 vs. 63.6 ± 10.4	11/10 vs. 22/11	I-III	General anesthesia with a single-shot ISB	The time to the first request for additional analgesic.	Y	20 ml of 0.25% levobupivacaine	dexamethasone 3.3 mg PN.	DOA/VAS
18	Sakae et al. (37)	2017	60	52.05 ± 13.7 vs. 52.1 ± 12.3 vs. 53.2 ± 9.8	11/9 vs. 14/6 vs. 12/8	I-II	General anesthesia with a single-shot ISB	The duration of the sensory block.	Y	20 ml of 0.75% ropivacaine	dexamethasone 4 mg PN; dexamethasone 4 mg I.V.	DOA/VAS
19	Yang et al. (38)	2019	87	59.2 ± 6.7 vs. 58.5 ± 8.0vs. 59.8 ± 7.6	19/10 vs. 21/8 vs. 17/12	I-II	General anesthesia with a single-shot ISB	The duration of the sensory block.	Y	20 ml of 0.5% ropivacaine	dexamethasone 0.05 mg/kg or 0.1 mg/kg I.V.	DOA/VAS
20	Jin and Wu (39)	2019	120	52.31 ± 12.9 vs. 53.5 ± 13.1vs. 53.5 ± 14.2	26/14 vs. 30/10vs. 32/8	I-II	General anesthesia with a single-shot ISB	The duration of the sensory block.	Y	20 ml of 0.75% ropivacaine	dexamethasone 2 mg PN; dexamethasone 4 mg I.V.	DOA/VAS
21	Lv et al. (40)	2020	75	50.2 ± 12.1 vs. 49.3 ± 14.3vs. 53.7 ± 11.0	15/10 vs. 14/11vs. 16/9	I-II	General anesthesia with a single-shot ISB	The duration of the sensory block.	Y	20 ml of 0.5% ropivacaine	dexamethasone 0.1mg/kg + dexmedetomidine 2 mcg/kg I.V.; dexamethasone 0.1 mg/kg I.V.	DOA/VAS
22	Shen and Chen (41)	2021	80	56.4 ± 14.3 vs. 55.7 ± 13.2	10/13 vs. 11/12	I-II	General anesthesia with a single-shot ISB	The duration of nerve block.	Y	20 ml of 0.375% ropivacaine	dexamethasone 5 mg PN.	DOA/VAS
23	Yu (42)	2021	70	55.73 ± 5.46 vs. 56.24 ± 5.97	21/14 vs. 22/13	/	General anesthesia with a single-shot ISB	The duration of the sensory block.	Y	20 ml of 0.5% ropivacaine	dexamethasone 0.10 mg/kg PN.	DOA
24	Qian et al. (43)	2018	40	56 ± 11 vs. 56 ±14	11/9 vs. 11/9	I-II	General anesthesia with a single-shot ISB	The time to the first request for additional analgesic.	Y	20 ml of 0.375% ropivacaine	dexmedetomidine 1 mcg/kg PN	DOA/VAS
25	Feng et al.	2021	30	42.2 ± 6.34 vs. 43.3 ± 5.14vs. 44.6 ± 10.0	7/3 vs. 6/4vs. 6/4	I-II	General anesthesia with a single-shot ISB	The duration of the sensory block.	Y	20 ml of 0.5% ropivacaine	dexamethasone 4 mg PN; dexmedetomidine 1 mcg/kg PN	DOA/VAS

RCT, Randomized controlled trial; CG, Control group. DXM, dexamethasone; DEX, dexmedetomidine; Y, yes; N, no; IV, intravenous; PN, perineural; DOA, duration of analgesia; O, opioid.

**Figure 2:**
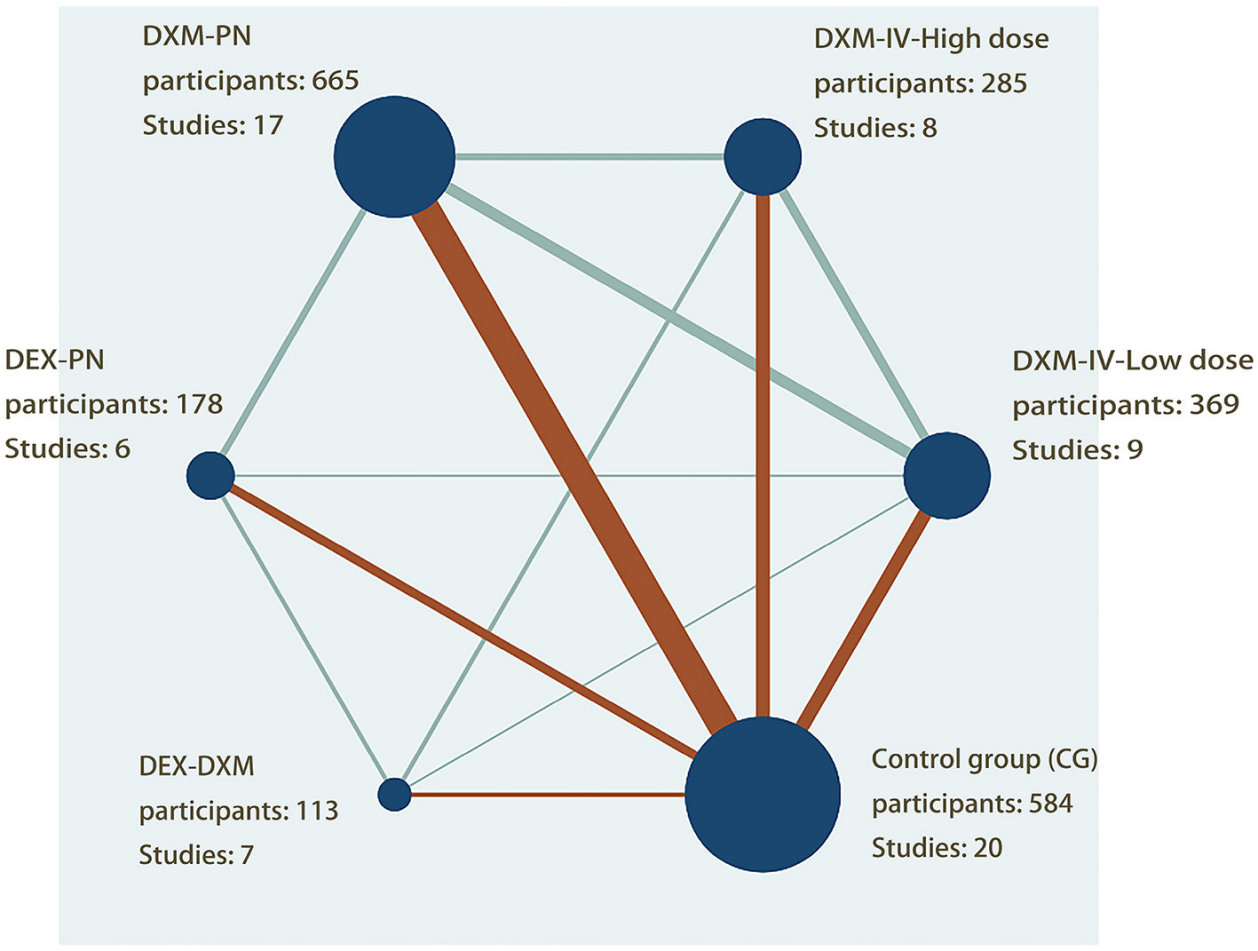
Network plot of evidence of all the trials. The network plot of the intervention network shows the comparion of the sample size to provide anesthesia for patients undergoing arthroscopic shoulder surgery. Each node represented a different method of prevention with the size of the node depending on the number of patients who received the intervention directly, he nodes were connected by lines indicating direct relationships between interventions, with the thickness of the line depending on the amount of direct evidence supporting the intervention.

A total of 2,194 patients who underwent arthroscopic shoulder surgery for arthroscopic rotator cuff, subacromial decompression, and various forms of shoulder surgery were enrolled in 25 studies published between 2013 and 2021 and included in the review. Five therapies were tested in parallel (*n* = 9) or crossover (*n* = 16) RCTs (1–4, 7, 25–44). The sample size was largest for the DXM-PN group (*n* = 665; 17 studies), followed by the control (*n* = 584; 20 studies), low-dose DXM-IV (*n* = 369; 9 studies), high-dose DXM-IV (*n* = 285; 8 studies), DEX-PN (*n* = 178; 6 studies), and DEX-DXM groups (*n* = 113; 3 studies). A network map was created to allow direct comparison between the interventions (Figure 2).

**Figure 3:**
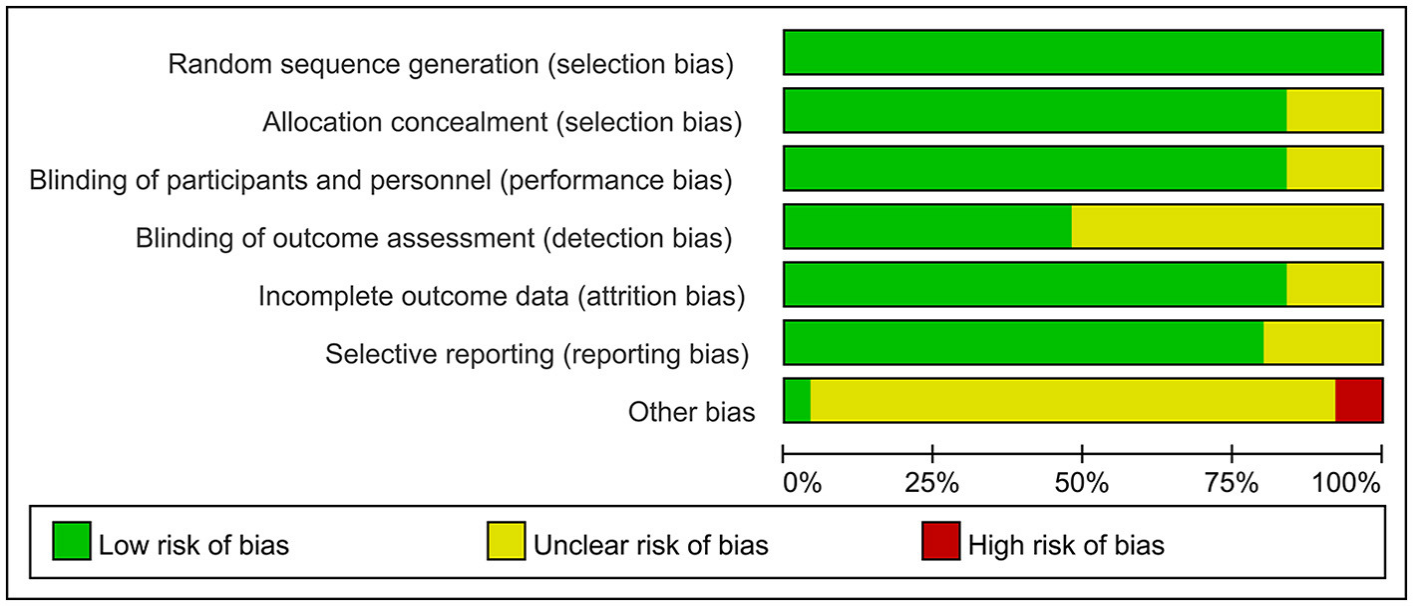
Risk of bias graph.

**Figure 4:**
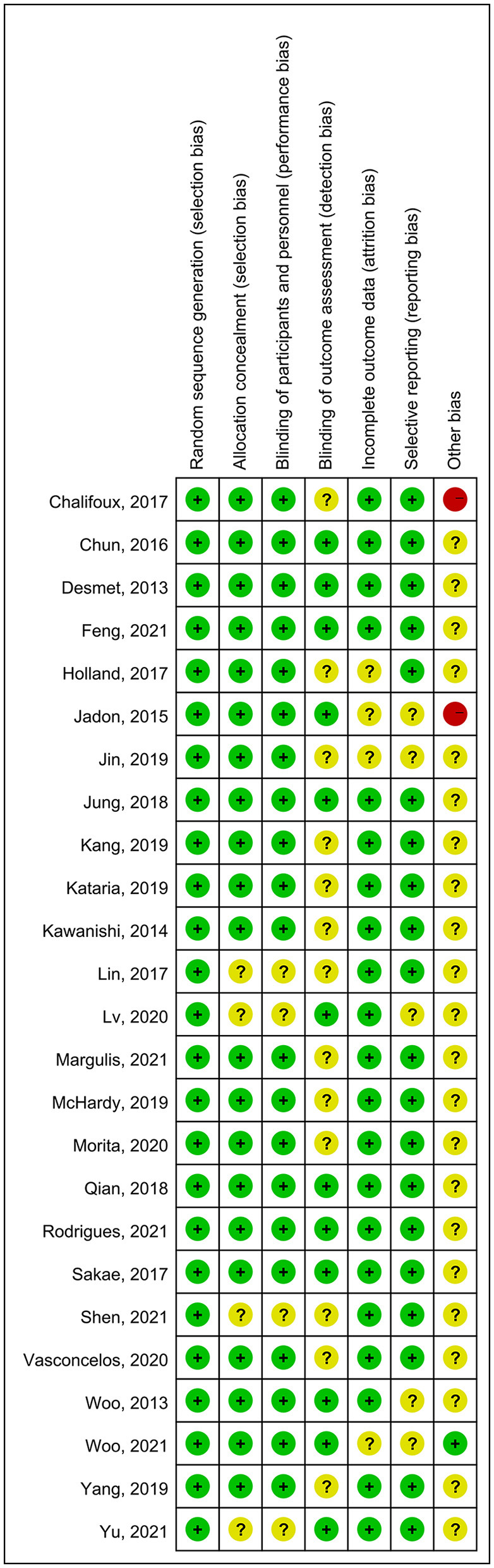
Risk of bias summary.

**Figure 5:**
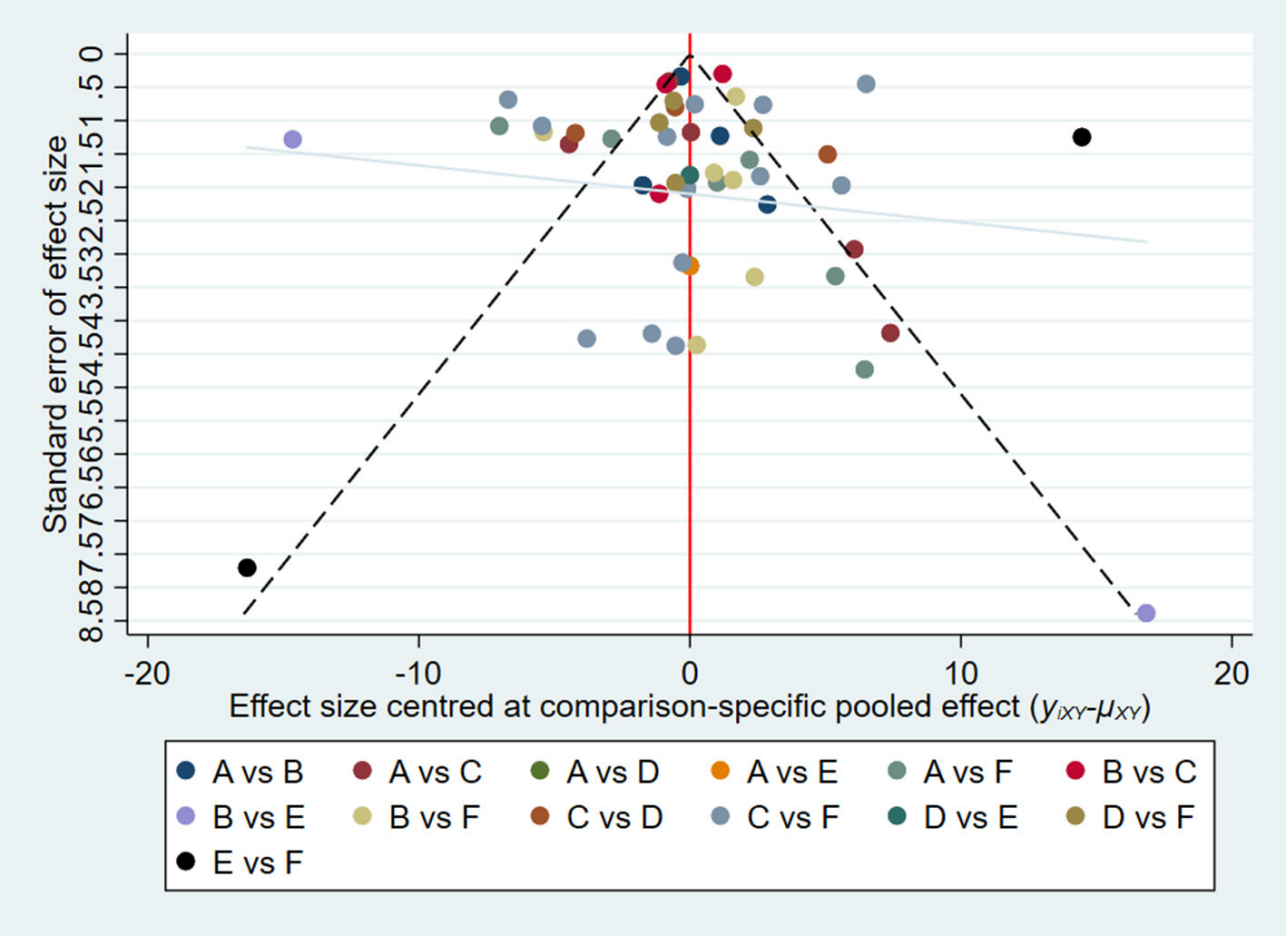
Funnel plot.

The overall quality of the included studies revealed modest variance. All 25 included trials were randomly allocated and showed a low risk of bias in “random sequence generation.” Twenty studies had a low ROB with selective outcome reporting. Four studies had a high or unclear risk for attrition bias. Twenty-one studies used allocation concealment, whereas 21 fully detailed the blinding of the outcome evaluation. Evaluation of the quality of the included studies is shown in Figures 3, 4. Publication bias was not observed in the funnel plot based on its symmetrical distribution (inverted funnel plot) (Figure 5). When consistency and inconsistency between studies were assessed, all *P*-values were > 0.05, showing that the effect of consistency between studies was acceptable. According to the *I*^2^ value, there was low to moderate heterogeneity among the included studies. Sensitive analysis by omitting one study in each turn indicated the results were unaffected. No single study notably affected the overall summary estimate and *P*-value. The details are shown in the Supplementary material.

### 3.2. Duration of analgesia

Twenty-five studies enrolling 2,194 patients reported the duration of analgesia. The placebo group included 584 patients, and the intervention group included 1610 (low-dose DXM-IV = 369, high-dose DXM-IV = 285, DXM-PN = 665, DEX-PN = 178, DEX-DXM = 113). Combined DEX-DXM (MD = 22.13, 95% CI 16.67, 27.58), DXM-PN (MD = 9.94, 95% CI 7.71, 12.17), high-dose DXM-IV (MD = 7.47, 95% CI 4.41, 10.53), DEX-PN (MD = 6.82, 95% CI 3.43, 10.20), and low-dose DXM-IV (MD = 6.72, 95% CI 3.74, 9.70) provided significantly longer analgesic effects compared with the control group.

According to the SUCRA data (Supplementary Figure 1), the combination of DEX-DXM (SUCRA = 98.5%) and DXM-PN (77.6%) had the highest efficacy, followed by high-dose DXM-IV (47.0%), DEX-PN (38.7%), low-dose DXM-IV (36.6%), and control groups (0.3%).

### 3.3. Opioids consumption

Eight studies enrolling a total of 939 patients reported opioid consumption after surgery. The placebo group included 110 patients, and the intervention group included 829 (low-dose DXM-IV = 248, high-dose DXM-IV = 116, DXM-PN = 252, DEX-PN = 125, DEX-DXM = 88). The DEX-DXM (MD = −4.50, 95% CI −5.25, −3.75), DXM-PN (MD = −4.70, 95% CI −5.53, −3.87), low-dose DXM-IV (MD = −30.03, 95% CI −46.35, −13.71), high-dose DXM-IV (MD =-4.50, 95% CI −5.28, −3.72), and DEX-PN (MD =-4.40, 95% CI −5.31, −3.49) groups had significantly better outcomes than the control group (Supplementary Figure 2).

The SUCRA data showed that the DEX-DXM group (SUCRA = 99.4%) had the highest efficacy, followed by the DXM-PN (SUCRA = 66.7%), low-dose DXM-IV (SUCRA = 47.7%), high-dose DXM-IV (SUCRA = 45.2%), DEX-PN (SUCRA = 41.0%), and control groups (0.5%).

### 3.4. Pain score

Sixteen studies enrolling a total of 1,307 patients reported pain scores (VAS or NRS) 24 h after surgery. The placebo group included 422 patients, and the intervention group included 885 (low-dose DXM-IV = 202, high-dose DXM-IV = 127, DXM-PN = 471, DEX-PN = 60, DEX-DXM = 25). The combined DEX-DXM (MD = −2.56, 95% CI −4.53, −0.59), high-dose DXM-IV (MD = −1.79, 95% CI −2.93, −0.66), DXM-PN (MD = −1.46, 95% CI −2.17, −0.75), and low-dose DXM-IV (MD = −1.06, 95% CI −2.08, −0.05) groups provided significantly longer analgesic effects than the control group (Supplementary Figure 3).

The SUCRA data showed that the DEX-DXM group (SUCRA = 89.3%) had the highest efficacy, followed by the high-dose DXM-IV (SUCRA = 72.9%), DXM-PN (SUCRA = 60.1%), low-dose DXM-IV (SUCRA = 39.8%), DEX-PN (SUCRA = 36.0%), and control groups (1.9%).

### 3.5. Adverse events

Two studies referred to transient paresthesias during block performance, 2 studies mentioned bradycardia, 4 studies have described hoarseness of voice, 4 studies pointed Horner' s syndrome, 3 studies mentioned dyspnea, 2 studies referred residual motor weakness, and 2 studies pointed numbness. Redness at the injection site, nerve injury, sleep disturbance and persistent distal surgical arm pain only mentioned in one study. Specific details of adverse events are summarized in Table 2.

**Table 2:** Adverse events of included studies.

**ID**	**Study**	**Total**	**Intervention**	**Adverse events**	**Summary**
1	Qian et al. (43)	40	Dexmedetomidine 0.1 mcg/kg I.V.	Four patients in DEX-PN group and three in CG experienced bradycardia during operation. There was no difference between groups.	bradycardia
2	Rodrigues et al. (25)	197	Dexmedetomidine 50 mcg PN., dexamethasone 4 mg I.V., dexmedetomidine 50 mcg + dexamethasone 4 mg I.V.	Four patients experienced transient paresthesias during block performance, one of whom had hoarseness and persistent distal surgical arm pain at 14 days but not 6 months postoperatively. An additional two patients in DEX-PN group experienced bradycardia during block performance.	transient paresthesias; hoarseness; arm pain; bradycardia
3	Holland et al. (7)	209	Dexamethasone 4 mg or 8 mg I.V. or 8 mg PN.	Four patients in DXM-PN group and one patient in high-dose DXM-IV group experienced transient paresthesias. The other patient experienced block-related pneumothorax. One other patient in DXM-PN group experienced dyspnoea.	transient paresthesias; pneumothorax; dyspnoea
4	Jung et al. (3)	47	Dexmedetomidine 2 mcg/kg I.V.	One patient experienced moderate dyspnea that resolved 18 h after ISB. Five patients in different groups experienced hypoxemia.	dyspnea; hypoxemia
5	Kataria et al. (28)	60	Dexmedetomidine 0.5 mcg/kg PN; dexamethasone 8 mg PN.	Horner's syndrome and hoarseness of voice were seen among different the groups.	horner's syndrome; hoarseness
6	Jadon et al. (30)	112	Dexamethasone 8 mg PN.	Eleven patients in DXM-PN group and 15 in CG experienced horner's syndrome. Ten patients in DXM-PN group and 8 in CG experienced ipsilateral diaphragmatic paresis. One patient in DXM-PN group and 2 in CG experienced hoarseness of voice. There were no differences in the incidence of horner's syndrome, hoarseness, and ipsilateral diaphragmatic after operation.	horner's syndrome; hoarseness; ipsilateral diaphragmatic paresis
7	Desmet et al. (31)	144	Dexamethasone 10 mg I.V.; dexamethasone 10 mg PN	There were no differences in the incidence of hoarseness, dyspnoea, or horner's syndrome after operation.	hoarseness; dyspnoea; horner's syndrome
8	Lin et al. (34)	60	Dexamethasone 0.05 mg/kg I.V., dexamethasone 0.1 mg/kg I.V.	Three patients in DXM-IV group experienced residual motor weakness, 8 experienced horner's syndrome and 2 experienced hoarseness of voice. One patient in CG experienced residual motor weakness, 3 experienced horner's syndrome in CG. There was no difference between groups	residual motor weakness; hoarseness; horner's syndrome
9	Chalifoux et al. (27)	69	Dexamethasone 4 mg I.V., dexamethasone 10 mg I.V.	A small proportion of patients experienced residual motor weakness at 24 and 48 h after surgery. There was no difference between groups	residual motor weakness
10	Chun et al. (45)	99	Dexamethasone 5 mg I.V., dexamethasone 5 mg PN.	One patient in high dose-DXM-IV group and 2 patients in DXM-PN group experienced numbness at 24 h after surgery.	numbness
11	Woo, et al. (26)	72	Dexamethasone 5 mg PN.	Three patients in DXM-PN group experienced arm numbness on the second day after surgery	numbness
12	Kawanishi et al. (4)	34	Dexamethasone 4 mg PN., dexamethasone 4 mg I.V.	One patient in low dose-DXM-IV group experienced redness at the injection site. This redness disappeared gradually, and the patient required no further therapy.	redness
13	Yang et al. (38)	87	Dexamethasone 0.05 mg/kg or 0.1 mg/kg I.V.	Three patients experienced nerve injury, but relevant details were not covered. There was no difference between groups.	nerve injury
14	Woo et al. (32)	70	Dexamethasone 5 mg PN.	During the first week postoperatively, 91.4% of patients in CG and 60% of patients in DXM-PN group experienced sleep disturbance at least once.	sleep disturbance

CG, Control group; DXM, dexamethasone; DEX, dexmedetomidine; I.V., intravenous; PN, perineural.

## 4. Discussion

In this study we assessed the relative efficacy of dexamethasone and dexmedetomidine as adjuncts to prolong the analgesic duration for a single- shot ISB. A total of 25 studies were included, including 2194 patients undergoing shoulder arthroscopy. Our study showed that the combination of intravenous dexamethasone and dexmedetomidine provided the greatest effect in terms of prolonged analgesia, reduced opioid doses, and lower pain scores. Furthermore, peripheral dexamethasone in prolonging the analgesic duration and lowering opioid usage was better than the other adjuvants when used a single medication. All therapies significantly prolonged the analgesic duration and reduced the opioid dose of a single-shot ISB in shoulder arthroscopy compared with the placebo.

Shoulder arthroscopy may cause considerable discomfort, particularly during the first 24 h following surgery. Several adjuvants, including intravenous dexamethasone, peripheral dexamethasone, peripheral dexmedetomidine, and the combined application of dexamethasone and dexmedetomidine, have been shown to extend the duration of nerve block (1). Our analysis quantitatively compared the effects of these adjuvants.

The exact mechanism by which dexamethasone prolongs the duration of the sensory blockade is unclear. Although glucocorticoids have been claimed to have direct effects on nerves, other investigations have indicated that dexamethasone may cause peripheral vasoconstriction and impede local anesthetic absorption (5). In a recent retrospective cohort analysis of upper and lower limb surgery under different forms of peripheral nerve block, intravenous dexamethasone was shown to extend the duration of the block when added to ropivacaine (46). Dexamethasone is also recognized as an auxiliary function in regional analgesia according to many single studies and a meta-analysis of 29 studies (47, 48). An RCT demonstrated that in patients who received ultrasound-guided sciatic nerve blocks, there was no significant difference between peripheral and intravenous dexamethasone in terms of the duration of analgesia (49). In major and small orthopedic surgeries, dexamethasone and other glucocorticoids have considerable analgesic effects in the equivalent dosage range of 9 to 40 mg dexamethasone (45, 50–52). In shoulder surgery, relatively little data are available, although dexamethasone (4–8 mg) has been used as an adjuvant for an ISB with a prolonged analgesic effect (53, 54). In addition, corticosteroid injections around the nerve have been utilized for a long time to treat radiculopathy. To date, no clinical studies have identified the neurological problems induced by dexamethasone (5).

Individual studies have shown that dexamethasone may extend the analgesic effect of ropivacaine when administered as an adjuvant; however, there are few direct head-to-head comparisons, and the findings are uncertain or even contradictory. A meta-analysis by Choi et al. involving 393 patients who received dexamethasone demonstrated that dexamethasone as a local anesthetic adjuvant lengthened the analgesic time of brachial plexus block (5). According to our results, peripheral dexamethasone is more efficient than intravenous dexamethasone. High-dose and low-dose intravenous dexamethasone were equivalent.

Dexmedetomidine is currently one of the most commonly used adjuvants for nerve blocking because it has no significant neurotoxicity risk. It is hypothesized that α_2_ receptor binding in the central nervous system mediates the analgesic effects of dexmedetomidine by decreasing the release of nociceptive transmitters (55). Brummett et al. first reported in 2008 that dexmedetomidine enhanced the duration of sciatic nerve block in rats without causing neurotoxicity (56). Several clinical trials have studied the beneficial effect of a single dose of peripheral dexmedetomidine on prolonging the analgesic time of nerve blocks (6, 57, 58). In a study conducted by Abdallah et al., intravenous or perineural administration of 0.5 mcg/kg dexmedetomidine was compared with the placebo. A total of 24h use of morphine after surgery was decreased in the dexmedetomidine group, but there was no significant difference in resting pain levels between the three groups after 24h (59). Our results are consistent with those of previous studies. Compared with the control group, dexmedetomidine prolonged the analgesic time of the brachial plexus block.

Our NMA demonstrates that the combination of dexamethasone and dexmedetomidine has the greatest analgesic effects in terms of prolonging analgesia. The mechanisms why dexamethasone could prolong analgesia are corticosteroid induced vasoconstriction reducing local anesthetic absorption and the inhibition of potassium channels on nociceptive C-fibers or inhibits inflammatory responses through peripheral and central (60–62). The synergistic mechanism why dexmedetomidine could prolong analgesia may be mediated by the binding of a2 receptors in the central nervous system, which inhibits the release of nociceptive transmitters (55, 63). The combined effects of the two drugs are usually antagonistic, additive, or synergistic (64). This mechanism can potentially be explained using the effect-addition model (65). However, the exact mechanism underlying the interaction between dexamethasone and dexmedetomidine need more studies to confirm.

In all of the studies we included, there was no significant difference in the incidence of adverse reactions between the groups using adjuvants or systemic medications compared to the control group. In addition, no matter what kind of adjuvant is used, there was no significant improvement in the incidence of complications related to nerve block treatments, such as dyspnea, hoarseness, and Horner's syndrome. As for peripheral dexamethasone or dexmedetomidine, studies have revealed that it is typically harmless (48, 66). In a recent study, data from 1026 individuals who received perineural dexmedetomidine were included, and it was shown that none of them experienced any neurotoxicity symptoms and neurologic sequalae (66). However, in patients with pre-existing heart disease, a systemic impact on the cardiovascular system remains a potential concern at high doses. Dexmedetomidine can cause bradycardia which may be the result of decreased central sympathetic output and increased parasympathetic output from cardiac vagal neurons in the brainstem (67). Hussain et al. (68) reported peripheral dexamethasone does not appear to lead to long-term neurologic complications and no persistent neurological deficits were reported in all included RCTs (68). Ma et al. (69) showed during *in-vitro* studies that dexamethasone may have a protective effect against local anesthetic-induced cell injury (69) and for the treatment of post-traumatic visual disturbance, a series of 2,000 intrathecal injections had no neurological sequelae (70). Some other evidences also suggest that dexamethasone may be neuroprotective, and it has been demonstrated that corticosteroids have no long-term electrophysiological, behavioral, or histological effects on the sciatic nerve tissue of rats (71). In general, the safety profile of perineural dexamethasone is promising.

This study had several limitations. First, it is difficult to evaluate the sensory blocks after surgery. Most studies use the time before the first pain relief as a sign of cessation of the sensory block. Other studies have only described the duration of analgesia or sensory blockade. Furthermore, the “off-label” use of adjuvants surrounding the nerve poses safety concerns. Without human clinical trials, we can only claim that there is no increase in neuronal cell death following exposure to low-dose dexamethasone plus ropivacaine for 2 h compared with ropivacaine alone based on laboratory investigations (72) and there is no neurological damage in perineural dexmedetomidine studies, as previously reported.

Our study has several advantages. When evaluating the prolonged analgesic effects of different adjuvants in the intermuscular sulcus brachial plexus, we restricted the surgery to the same type. Only RCT studies were included in our analysis, and the quality assessment results of all publications were similar.

In conclusion, the combination of intravenous dexamethasone and dexmedetomidine provided the greatest effect in terms of prolonged analgesia, reduced opioid doses, and lower pain scores. Furthermore, peripheral dexamethasone in prolonging the analgesic duration and lowering opioid usage was better than the other adjuvants when used a single medication. All therapies significantly prolonged the analgesic duration and reduced the opioid dose of a single-shot ISB in shoulder arthroscopy compared with the placebo.

## Data availability statement

The original contributions presented in the study are included in the article/Supplementary material, further inquiries can be directed to the corresponding author.

## Author contributions

X-MW and ZL provided substantial contributions to the conception or design of the work, acquisition, analysis, interpretation of the data, drafting of the manuscript, and critically revising the manuscript for important intellectual content. L-CL contributed substantially to the conception and design of the work, acquisition, analysis, interpretation of the data, drafting of the manuscript, and critically revising the manuscript for important intellectual content. G-HW agreed to be accountable for all aspects of the work in ensuring that questions related to the accuracy or integrity of any part of the work are appropriately investigated and resolved. P-YS contributed substantially to the revision of the work and agreed to be accountable for all aspects of the work in ensuring that questions related to the accuracy or integrity of any part of the work are appropriately investigated and resolved. C-PG helped with the approval of the final version of the manuscript to be published. P-CS helped with the approval of the final version of the manuscript to be published. All authors contributed to the article and approved the submitted version.
