# Supplementary material for: Comparison of dexmedetomidine and dexamethasone as adjuvants to the ultrasound-guided interscalene nerve block in arthroscopic shoulder surgery: a systematic review and Bayesian network meta-analysis of randomized controlled trials
Source: Front Med (Lausanne). 2023 Jun 16;10:1159216. doi: 10.3389/fmed.2023.1159216 (PMC10312098; doi:10.3389/fmed.2023.1159216)
Supplement: Supplementary file 3 [file Data_Sheet_3.pdf]

PubMed: 185

#1 "arthroscopy"[MeSH Terms]

#2 "arthroscopy"[Title/Abstract]

#3 "shoulder"[MeSH Terms]

#4 "shoulder"[Title/Abstract]

#5 "arthroscopy"[MeSH Terms] OR "arthroscopy"[Title/Abstract]

#6 "shoulder"[MeSH Terms] OR "shoulder"[Title/Abstract]

#7 ("arthroscopy"[MeSH Terms] OR "arthroscopy"[Title/Abstract]) AND ("shoulder"[MeSH Terms] OR "shoulder"[Title/Abstract])

#8 ("arthroscoped"[All Fields] OR "arthroscopes"[MeSH Terms] OR "arthroscopes"[All Fields] OR "arthroscope"[All Fields] OR "arthroscopic"[All Fields] OR "arthroscopical"[All Fields] OR "arthroscopically"[All Fields]) AND ("shoulder"[MeSH Terms] OR "shoulder"[All Fields] OR "shoulders"[All Fields] OR "shoulder s"[All Fields]) AND ("surgery"[MeSH Subheading] OR "surgery"[All Fields] OR "surgical procedures, operative"[MeSH Terms] OR ("surgical"[All Fields] AND "procedures"[All Fields] AND "operative"[All Fields]) OR "operative surgical procedures"[All Fields] OR "general surgery"[MeSH Terms] OR ("general"[All Fields] AND "surgery"[All Fields]) OR "general surgery"[All Fields] OR "surgery s"[All Fields] OR "surgerys"[All Fields] OR "surgeries"[All Fields])

#9 (("arthroscopy"[MeSH Terms] OR "arthroscopy"[Title/Abstract]) AND ("shoulder"[MeSH Terms] OR "shoulder"[Title/Abstract])) OR (("arthroscoped"[All Fields] OR "arthroscopes"[MeSH Terms] OR "arthroscopes"[All Fields] OR "arthroscope"[All Fields] OR "arthroscopic"[All Fields] OR "arthroscopical"[All Fields] OR "arthroscopically"[All Fields]) AND ("shoulder"[MeSH Terms] OR "shoulder"[All Fields] OR "shoulders"[All Fields] OR "shoulder s"[All Fields]) AND ("surgery"[MeSH Subheading] OR "surgery"[All Fields] OR "surgical procedures, operative"[MeSH Terms] OR ("surgical"[All Fields] AND "procedures"[All Fields] AND "operative"[All Fields]) OR "operative surgical procedures"[All Fields] OR "general surgery"[MeSH Terms] OR ("general"[All Fields] AND "surgery"[All Fields]) OR "general surgery"[All Fields] OR "surgery s"[All Fields] OR "surgerys"[All Fields] OR "surgeries"[All Fields]))

#10 "nerve block"[MeSH Terms]

#11 "nerve block"[Title/Abstract]

#12 "anesthesia, local"[MeSH Terms]

#13 "anesthesia local"[Title/Abstract]

#14 "anesthesia, conduction"[MeSH Terms]

#15 "regional anesthesia"[Title/Abstract]

#16 "local anesthesia"[Title/Abstract]

#17 "regional block"[Title/Abstract]

#18 "interscalene nerve block"[Title/Abstract]

#19 ("interscalene"[All Fields] OR "interscalenic"[All Fields]) AND ("nerve block"[MeSH Terms] OR ("nerve"[All Fields] AND "block"[All Fields]) OR

"nerve block"[All Fields])

#20 "suprascapular nerve block"[Title/Abstract]

#21 "suprascapular"[All Fields] AND ("nerve block"[MeSH Terms] OR ("nerve"[All Fields] AND "block"[All Fields]) OR "nerve block"[All Fields])

#22 "supraclavicular nerve block"[Title/Abstract]

#23 "supraclavicular"[All Fields] AND ("nerve block"[MeSH Terms] OR ("nerve"[All Fields] AND "block"[All Fields]) OR "nerve block"[All Fields])

#24 "suprascapular"[Title/Abstract] AND "axillary nerve blocks"[Title/Abstract]

#25 "suprascapular"[All Fields] AND ("axilla"[MeSH Terms] OR "axilla"[All Fields] OR "axillary"[All Fields] OR "axillaries"[All Fields] OR "axillaris"[All Fields]) AND ("nerve block"[MeSH Terms] OR ("nerve"[All Fields] AND "block"[All Fields]) OR "nerve block"[All Fields] OR ("nerve"[All Fields] AND "blocks"[All Fields]) OR "nerve blocks"[All Fields])

#26 "nerve block"[MeSH Terms] OR "nerve block"[Title/Abstract]

#27 "anesthesia, local"[MeSH Terms] OR "anesthesia local"[Title/Abstract]

#28 "anesthesia, conduction"[MeSH Terms] OR "regional anesthesia"[Title/Abstract]

#29 "interscalene nerve block"[Title/Abstract] OR (("Interscalene"[All Fields] OR "interscalenic"[All Fields]) AND ("nerve block"[MeSH Terms] OR ("nerve"[All Fields] AND "block"[All Fields]) OR "nerve block"[All Fields]))

#30 "suprascapular nerve block"[Title/Abstract] OR ("suprascapular"[All Fields] AND ("nerve block"[MeSH Terms] OR ("nerve"[All Fields] AND "block"[All Fields]) OR "nerve block"[All Fields]))

#31 "supraclavicular nerve block"[Title/Abstract] OR ("supraclavicular"[All Fields] AND ("nerve block"[MeSH Terms] OR ("nerve"[All Fields] AND "block"[All Fields]) OR "nerve block"[All Fields]))

#32 ("suprascapular"[Title/Abstract] AND "axillary nerve blocks"[Title/Abstract]) OR ("suprascapular"[All Fields] AND ("axilla"[MeSH Terms] OR "axilla"[All Fields] OR "axillary"[All Fields] OR "axillaries"[All Fields] OR "axillaris"[All Fields]) AND ("nerve block"[MeSH Terms] OR ("nerve"[All Fields] AND "block"[All Fields]) OR "nerve block"[All Fields] OR ("nerve"[All Fields] AND "blocks"[All Fields]) OR "nerve blocks"[All Fields]))

#33 "continuous interscalene nerve block"[Title/Abstract]

#34 ("continual"[All Fields] OR "continually"[All Fields] OR "continuance"[All Fields] OR "continuation"[All Fields] OR "continuations"[All Fields] OR "continue"[All Fields] OR "continued"[All Fields] OR "continuer"[All Fields] OR "continuers"[All Fields] OR "continues"[All Fields] OR "continuing"[All Fields] OR "continuities"[All Fields] OR "continuity"[All Fields] OR "continuous"[All Fields] OR "continuously"[All Fields]) AND ("interscalene"[All Fields] OR "interscalenic"[All Fields]) AND ("nerve block"[MeSH Terms] OR ("nerve"[All Fields] AND "block"[All Fields]) OR "nerve block"[All Fields])

#35 "continuous interscalene nerve block"[Title/Abstract] OR (("continual"[All Fields] OR "continually"[All Fields] OR "continuance"[All Fields] OR "continuation"[All Fields] OR "continuations"[All Fields] OR "continue"[All

Fields] OR "continued"[All Fields] OR "continuer"[All Fields] OR "continuers"[All Fields] OR "continues"[All Fields] OR "continuing"[All Fields] OR "continuities"[All Fields] OR "continuity"[All Fields] OR "continuous"[All Fields] OR "continuously"[All Fields]) AND ("interscalene"[All Fields] OR "interscalenic"[All Fields]) AND ("nerve block"[MeSH Terms] OR ("nerve"[All Fields] AND "block"[All Fields]) OR "nerve block"[All Fields]))

#36 "nerve block"[MeSH Terms] OR "nerve block"[Title/Abstract] OR ("anesthesia, local"[MeSH Terms] OR "anesthesia local"[Title/Abstract]) OR ("anesthesia, conduction"[MeSH Terms] OR "regional anesthesia"[Title/Abstract]) OR "local anesthesia"[Title/Abstract] OR "regional block"[Title/Abstract] OR ("interscalene nerve block"[Title/Abstract] OR (("Interscalene"[All Fields] OR "interscalenic"[All Fields]) AND ("nerve block"[MeSH Terms] OR ("nerve"[All Fields] AND "block"[All Fields]) OR "nerve block"[All Fields]))) OR ("suprascapular nerve block"[Title/Abstract] OR ("suprascapular"[All Fields] AND ("nerve block"[MeSH Terms] OR ("nerve"[All Fields] AND "block"[All Fields]) OR "nerve block"[All Fields]))) OR ("supraclavicular nerve block"[Title/Abstract] OR ("supraclavicular"[All Fields] AND ("nerve block"[MeSH Terms] OR ("nerve"[All Fields] AND "block"[All Fields]) OR "nerve block"[All Fields]))) OR (("suprascapular"[Title/Abstract] AND "axillary nerve blocks"[Title/Abstract]) OR ("suprascapular"[All Fields] AND ("axilla"[MeSH Terms] OR "axilla"[All Fields] OR "axillary"[All Fields] OR "axillaries"[All Fields] OR "axillaris"[All Fields]) AND ("nerve block"[MeSH Terms] OR ("nerve"[All Fields] AND "block"[All Fields]) OR "nerve block"[All Fields] OR ("nerve"[All Fields] AND "blocks"[All Fields]) OR "nerve blocks"[All Fields]))) OR ("continuous interscalene nerve block"[Title/Abstract] OR (("continual"[All Fields] OR "continually"[All Fields] OR "continuance"[All Fields] OR "continuation"[All Fields] OR "continuations"[All Fields] OR "continue"[All Fields] OR "continued"[All Fields] OR "continuer"[All Fields] OR "continuers"[All Fields] OR "continues"[All Fields] OR "continuing"[All Fields] OR "continuities"[All Fields] OR "continuity"[All Fields] OR "continuous"[All Fields] OR "continuously"[All Fields]) AND ("Interscalene"[All Fields] OR "interscalenic"[All Fields]) AND ("nerve block"[MeSH Terms] OR ("nerve"[All Fields] AND "block"[All Fields]) OR "nerve block"[All Fields])))

#37 ("randomized controlled trial"[Publication Type] OR "controlled clinical trial"[Publication Type] OR "randomized"[Title/Abstract] OR "placebo"[Title/Abstract] OR "clinical trials as topic"[MeSH Terms:noexp] OR "randomly"[Title/Abstract] OR "trial"[Title]) NOT ("animals"[MeSH Terms] NOT ("humans"[MeSH Terms] AND "animals"[MeSH Terms]))

#38 ((("arthroscopy"[MeSH Terms] OR "arthroscopy"[Title/Abstract]) AND ("shoulder"[MeSH Terms] OR "shoulder"[Title/Abstract])) OR (("arthroscoped"[All Fields] OR "arthroscopes"[MeSH Terms] OR "arthroscopes"[All Fields] OR "arthroscope"[All Fields] OR "arthroscopic"[All Fields] OR "arthroscopical"[All Fields] OR "arthroscopically"[All Fields]) AND

("shoulder"[MeSH Terms] OR "shoulder"[All Fields] OR "shoulders"[All Fields] OR "shoulder s"[All Fields]) AND ("surgery"[MeSH Subheading] OR "surgery"[All Fields] OR "surgical procedures, operative"[MeSH Terms] OR ("surgical"[All Fields] AND "procedures"[All Fields] AND "operative"[All Fields]) OR "operative surgical procedures"[All Fields] OR "general surgery"[MeSH Terms] OR ("general"[All Fields] AND "surgery"[All Fields]) OR "general surgery"[All Fields] OR "surgery s"[All Fields] OR "surgerys"[All Fields] OR "surgeries"[All Fields]))) AND ("nerve block"[MeSH Terms] OR "nerve block"[Title/Abstract] OR ("anesthesia, local"[MeSH Terms] OR "anesthesia local"[Title/Abstract]) OR ("anesthesia, conduction"[MeSH Terms] OR "regional anesthesia"[Title/Abstract]) OR "local anesthesia"[Title/Abstract] OR "regional block"[Title/Abstract] OR ("interscalene nerve block"[Title/Abstract] OR (("Interscalene"[All Fields] OR "interscalenic"[All Fields]) AND ("nerve block"[MeSH Terms] OR ("nerve"[All Fields] AND "block"[All Fields]) OR "nerve block"[All Fields]))) OR ("suprascapular nerve block"[Title/Abstract] OR ("suprascapular"[All Fields] AND ("nerve block"[MeSH Terms] OR ("nerve"[All Fields] AND "block"[All Fields]) OR "nerve block"[All Fields]))) OR ("supraclavicular nerve block"[Title/Abstract] OR ("supraclavicular"[All Fields] AND ("nerve block"[MeSH Terms] OR ("nerve"[All Fields] AND "block"[All Fields]) OR "nerve block"[All Fields]))) OR (("suprascapular"[Title/Abstract] AND "axillary nerve blocks"[Title/Abstract]) OR ("suprascapular"[All Fields] AND ("axilla"[MeSH Terms] OR "axilla"[All Fields] OR "axillary"[All Fields] OR "axillaries"[All Fields] OR "axillaris"[All Fields]) AND ("nerve block"[MeSH Terms] OR ("nerve"[All Fields] AND "block"[All Fields]) OR "nerve block"[All Fields] OR ("nerve"[All Fields] AND "blocks"[All Fields]) OR "nerve blocks"[All Fields]))) OR ("continuous interscalene nerve block"[Title/Abstract] OR (("continual"[All Fields] OR "continually"[All Fields] OR "continuance"[All Fields] OR "continuation"[All Fields] OR "continuations"[All Fields] OR "continue"[All Fields] OR "continued"[All Fields] OR "continuer"[All Fields] OR "continuers"[All Fields] OR "continues"[All Fields] OR "continuing"[All Fields] OR "continuities"[All Fields] OR "continuity"[All Fields] OR "continuous"[All Fields] OR "continuously"[All Fields]) AND ("Interscalene"[All Fields] OR "interscalenic"[All Fields]) AND ("nerve block"[MeSH Terms] OR ("nerve"[All Fields] AND "block"[All Fields]) OR "nerve block"[All Fields])))) AND (("randomized controlled trial"[Publication Type] OR "controlled clinical trial"[Publication Type] OR "randomized"[Title/Abstract] OR "placebo"[Title/Abstract] OR "clinical trials as topic"[MeSH Terms:noexp] OR "randomly"[Title/Abstract] OR "trial"[Title]) NOT ("animals"[MeSH Terms] NOT ("humans"[MeSH Terms] AND "animals"[MeSH Terms])))

EMbase: 322

#1 'arthroscopy'/exp OR arthroscopy

#2 'shoulder'/exp OR shoulder

#3 ('arthroscopy'/exp OR arthroscopy) AND ('shoulder'/exp OR shoulder)

#4 arthroscopic shoulder surgery

#5 ('arthroscopy'/exp OR arthroscopy) AND ('shoulder'/exp OR shoulder) OR arthroscopic shoulder surgery

#6 'nerve block'/exp OR 'nerve block'

#7 'local anesthesia'/exp OR 'local anesthesia'

#8 'regional anesthesia'/exp OR 'regional anesthesia'

#9 'regional block' OR (regional AND block)

#10 'interscalene nerve block'/exp OR 'interscalene nerve block'

#11 'suprascapular nerve block'/exp OR 'suprascapular nerve block'

#12 'supraclavicular nerve block'/exp OR 'supraclavicular nerve block'

#13 suprascapular AND ('axillary nerve block'/exp OR 'axillary nerve block')

#14 'continuous interscalene nerve block' OR (continuous AND interscalene AND ('nerve'/exp OR nerve) AND block)

#15 'nerve block'/exp OR 'nerve block' OR 'local anesthesia'/exp OR 'local anesthesia' OR 'regional anesthesia'/exp OR 'regional anesthesia' OR 'regional block' OR (regional AND block) OR 'interscalene nerve block'/exp OR 'interscalene nerve block' OR 'suprascapular nerve block'/exp OR 'suprascapular nerve block' OR 'supraclavicular nerve block'/exp OR 'supraclavicular nerve block' OR (suprascapular AND ('axillary nerve block'/exp OR 'axillary nerve block')) OR 'continuous interscalene nerve block' OR (continuous AND interscalene AND ('nerve'/exp OR nerve) AND block)

#16 'crossover procedure':de OR 'double-blind procedure':de OR 'randomized controlled trial':de OR 'single-blind procedure':de OR random*:de,ab,ti OR factorial*:de,ab,ti OR crossover*:de,ab,ti OR ((cross NEXT/1 over*):de,ab,ti) OR placebo*:de,ab,ti OR ((doubl* NEAR/1 blind*):de,ab,ti) OR ((singl* NEAR/1 blind*):de,ab,ti) OR assign*:de,ab,ti OR allocat*:de,ab,ti OR volunteer*:de,ab,ti

#17 (('arthroscopy'/exp OR arthroscopy) AND ('shoulder'/exp OR shoulder) OR arthroscopic shoulder surgery) AND ('nerve block'/exp OR 'nerve block' OR 'local anesthesia'/exp OR 'local anesthesia' OR 'regional anesthesia'/exp OR 'regional anesthesia' OR 'regional block' OR (regional AND block) OR 'interscalene nerve block'/exp OR 'interscalene nerve block' OR 'suprascapular nerve block'/exp OR 'suprascapular nerve block' OR 'supraclavicular nerve block'/exp OR 'supraclavicular nerve block' OR (suprascapular AND ('axillary nerve block'/exp OR 'axillary nerve block')) OR 'continuous interscalene nerve block' OR (continuous AND interscalene AND ('nerve'/exp OR nerve) AND block)) AND ('crossover procedure':de OR 'double-blind procedure':de OR 'randomized controlled trial':de OR 'single-blind procedure':de OR random*:de,ab,ti OR factorial*:de,ab,ti OR crossover*:de,ab,ti OR ((cross NEXT/1 over*):de,ab,ti) OR placebo*:de,ab,ti OR ((doubl* NEAR/1

blind*):de,ab,ti) OR ((singl* NEAR/1 blind*):de,ab,ti) OR assign*:de,ab,ti OR allocat*:de,ab,ti OR volunteer*:de,ab,ti)

Cochrane: 417

#1 arthroscopy
#2 shoulder
#3 #1 and #2
#4 arthroscopic shoulder surgery
#5 #3 or #4
#6 nerve block
#7 MeSH descriptor: [Nerve Block] explode all trees
#8 local anesthesia
#9 MeSH descriptor: [Anesthesia, Local] explode all trees
#10 regional anesthesia
#11 MeSH descriptor: [Anesthesia, Conduction] explode all trees
#12 regional block
#13 interscalene nerve block
#14 suprascapular nerve block
#15 supraclavicular nerve block
#16 axillary nerve block
#17 continuous interscalene nerve block
#18 #6 or #7 or #8 or #9 or #10 or #11 or #12 or #13 or #14 or #15 or #16 or #17
#19 #5 and #18

Web Of Science: 424

#1 (TS=(arthroscopy)) OR TI=(arthroscopy)
#2 (TS=(shoulder)) OR TI=(shoulder)
#3 #1 and #2
#4 (TS=(arthroscopic shoulder surgery)) OR TI=(arthroscopic shoulder surgery)
#5 #3 Or #4
#6 (TS=(nerve block)) OR TI=(nerve block)
#7 (TS=(local anesthesia)) OR TI=(local anesthesia)
#8 (TS=(regional anesthesia)) OR TI=(regional anesthesia)
#9 (TS=(regional block)) OR TI=(regional block)
#10 (TS=(interscalene nerve block)) OR TS=(interscalene nerve block)
#11 (TS=(suprascapular nerve block)) OR TI=(suprascapular nerve block)
#12 (TI=(supraclavicular nerve block)) OR TS=(supraclavicular nerve block)
#13 (TS=(axillary nerve block)) OR TI=(axillary nerve block)
#14 (TS=(continuous interscalene nerve block)) OR TI=(continuous interscalene nerve block)
#15 #6 or #7 or #8 or #9 or #10 or #11 or #12 or #13 or #14
#16 #5 and #15
#17 TS= clinical trial* OR TS=research design OR TS=comparative stud* OR TS=evaluation stud* OR TS=controlled trial* OR TS=follow-up stud* OR

TS=prospective stud* OR TS=random* OR TS=placebo* OR TS=(single blind*) OR TS=(double blind*)

#18 #16 and #17
