# Supplementary material for: Comparison of dexmedetomidine and dexamethasone as adjuvants to the ultrasound-guided interscalene nerve block in arthroscopic shoulder surgery: a systematic review and Bayesian network meta-analysis of randomized controlled trials
Source: Front Med (Lausanne). 2023 Jun 16;10:1159216. doi: 10.3389/fmed.2023.1159216 (PMC10312098; doi:10.3389/fmed.2023.1159216)
Supplement: Supplementary file 4 [file Data_Sheet_4.pdf]

```
network meta i,force
Command is: mvmeta _y _S    , bscovariance(exch 0.5) longparm suppress(uv mm) eq(_y_B: des_ABF, _y_C: des_AC de
> s_ACF des_BC des_BCF, _y_D: des_CD des_CDF des_DF, _y_E: des_BEF, _y_F: des_ACF des_BCF des_BEF des_CDF des
> _CF) vars(_y_B _y_C _y_D _y_E _y_F)
Note: using method reml
Note: regressing _y_B on des_ABF
Note: regressing _y_C on des_AC des_ACF des_BC des_BCF
Note: regressing _y_D on des_CD des_CDF des_DF
Note: regressing _y_E on des_BEF
Note: regressing _y_F on des_ACF des_BCF des_BEF des_CDF des_CF
Note: 25 observations on 5 variables
Note: variance-covariance matrix is proportional to .5*I(5)+.5*J(5,5,1)

initial:       log likelihood = -210.77323
rescale:       log likelihood = -210.77323
rescale eq:    log likelihood = -171.14396
Iteration 0:   log likelihood = -171.14396
Iteration 1:   log likelihood = -170.16214
Iteration 2:   log likelihood = -170.10944
Iteration 3:   log likelihood = -170.10931
Iteration 4:   log likelihood = -170.10931

Multivariate meta-analysis
Variance-covariance matrix = proportional .5*I(5)+.5*J(5,5,1)
Method = reml                                   Number of dimensions    =      5
Restricted log likelihood = -170.10931          Number of observations  =     25
------------------------------------------------------------------------------
             |      Coef.   Std. Err.      z    P>|z|     [95% Conf. Interval]
-------------+----------------------------------------------------------------
_y_B         |
     des_ABF |   1.066942   4.996549     0.21   0.831    -8.726115       10.86
       _cons |   .7965718   4.231469     0.19   0.851    -7.496955    9.090098
-------------+----------------------------------------------------------------
_y_C         |
      des_AC |  -3.693974    6.13101    -0.60   0.547    -15.71053    8.322586
     des_ACF |   4.492701   5.142769     0.87   0.382     -5.58694    14.57234
      des_BC |   2.102527   5.992244     0.35   0.726    -9.642055    13.84711
     des_BCF |  -.2374728   6.341672    -0.04   0.970    -12.66692    12.19198
       _cons |   3.193974   4.236305     0.75   0.451    -5.109031    11.49698
-------------+----------------------------------------------------------------
_y_D         |
      des_CD |   8.593689   8.018459     1.07   0.284    -7.122202    24.30958
```

```
     des_CDF |   9.469781    7.461158     1.27   0.204    -5.153819    24.09338
      des_DF |   7.022356    6.620765     1.06   0.289    -5.954106    19.99882
       _cons |       -8.5    5.282469    -1.61   0.108    -18.85345    1.853449
-------------+----------------------------------------------------------------
_y_E         |
     des_BEF |   23.61949    7.946441     2.97   0.003     8.044754    39.19423
       _cons |        -.5    5.286093    -0.09   0.925    -10.86055    9.860552
-------------+----------------------------------------------------------------
_y_F         |
     des_ACF |   7.267263    3.981003     1.83   0.068    -.5353608    15.06989
     des_BCF |   .2952439     7.83535     0.04   0.970    -15.06176    15.65225
     des_BEF |   1.282555    5.973154     0.21   0.830    -10.42461    12.98972
     des_CDF |   4.210616    5.899458     0.71   0.475    -7.352109    15.77334
      des_CF |   1.220349    5.243232     0.23   0.816    -9.056198    11.49689
       _cons |  -8.138743    2.576708    -3.16   0.002      -13.189   -3.088489
------------------------------------------------------------------------------

Estimated between-studies SDs and correlation matrix:
              SD        _y_B        _y_C        _y_D        _y_E        _y_F
_y_B   4.2204346           1           .           .           .           .
_y_C   4.2204346          .5           1           .           .           .
_y_D   4.2204346          .5          .5           1           .           .
_y_E   4.2204346          .5          .5          .5           1           .
_y_F   4.2204346          .5          .5          .5          .5           1

Testing for inconsistency:
 ( 1)  [_y_B]des_ABF = 0
 ( 2)  [_y_C]des_AC = 0
 ( 3)  [_y_C]des_ACF = 0
 ( 4)  [_y_F]des_ACF = 0
 ( 5)  [_y_C]des_BC = 0
 ( 6)  [_y_C]des_BCF = 0
 ( 7)  [_y_F]des_BCF = 0
 ( 8)  [_y_F]des_BEF = 0
 ( 9)  [_y_E]des_BEF = 0
 (10)  [_y_D]des_CD = 0
 (11)  [_y_F]des_CDF = 0
 (12)  [_y_D]des_CDF = 0
 (13)  [_y_F]des_CF = 0
 (14)  [_y_D]des_DF = 0

           chi2( 14) =   20.11
         Prob > chi2 =    0.1267
mvmeta command stored as F9; test command stored as F8
```
