# Supplementary material for: Comparison of dexmedetomidine and dexamethasone as adjuvants to the ultrasound-guided interscalene nerve block in arthroscopic shoulder surgery: a systematic review and Bayesian network meta-analysis of randomized controlled trials
Source: Front Med (Lausanne). 2023 Jun 16;10:1159216. doi: 10.3389/fmed.2023.1159216 (PMC10312098; doi:10.3389/fmed.2023.1159216)
Supplement: Supplementary file 5 [file Data_Sheet_5.pdf]

Heterogeneity analysis

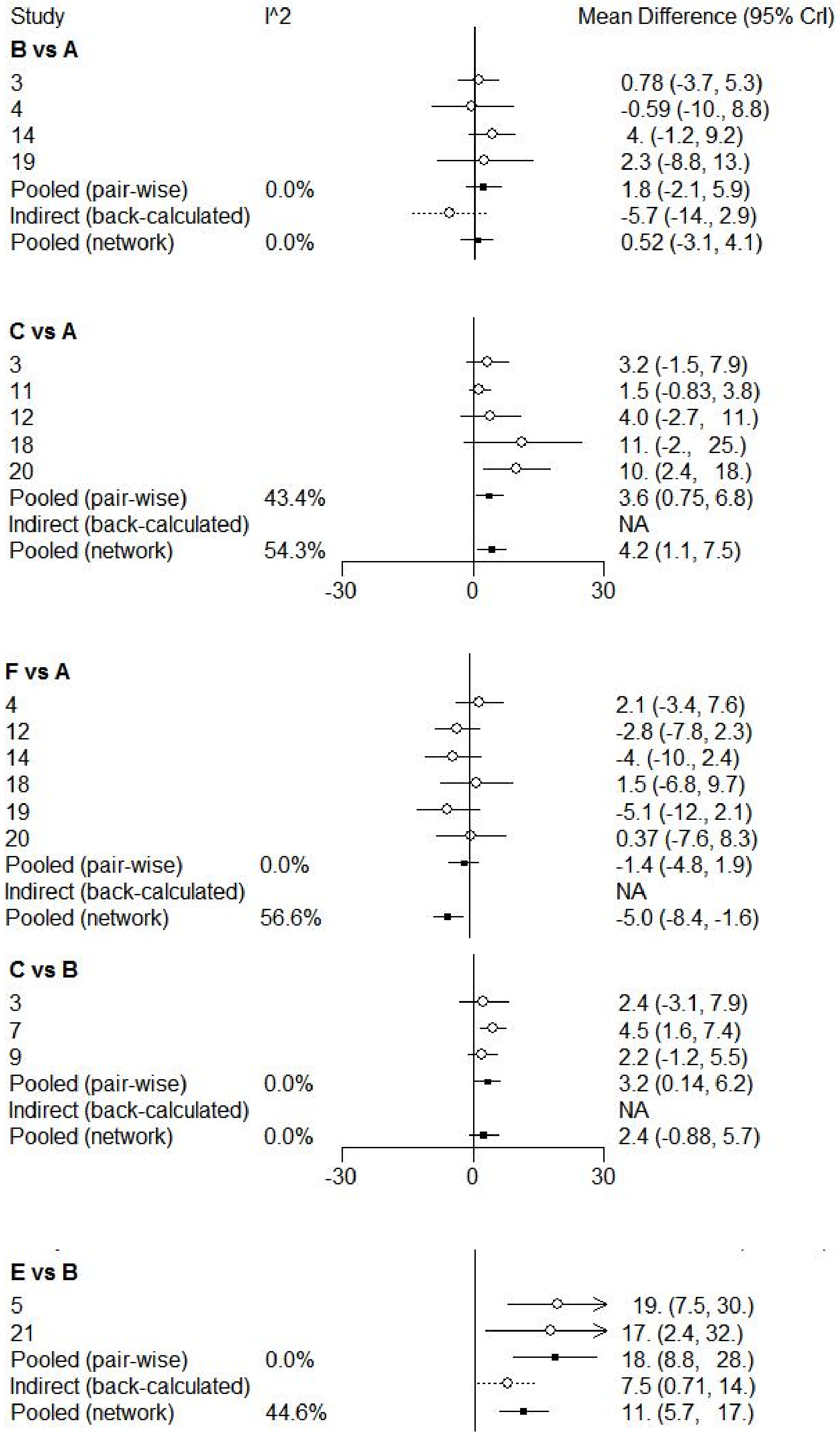

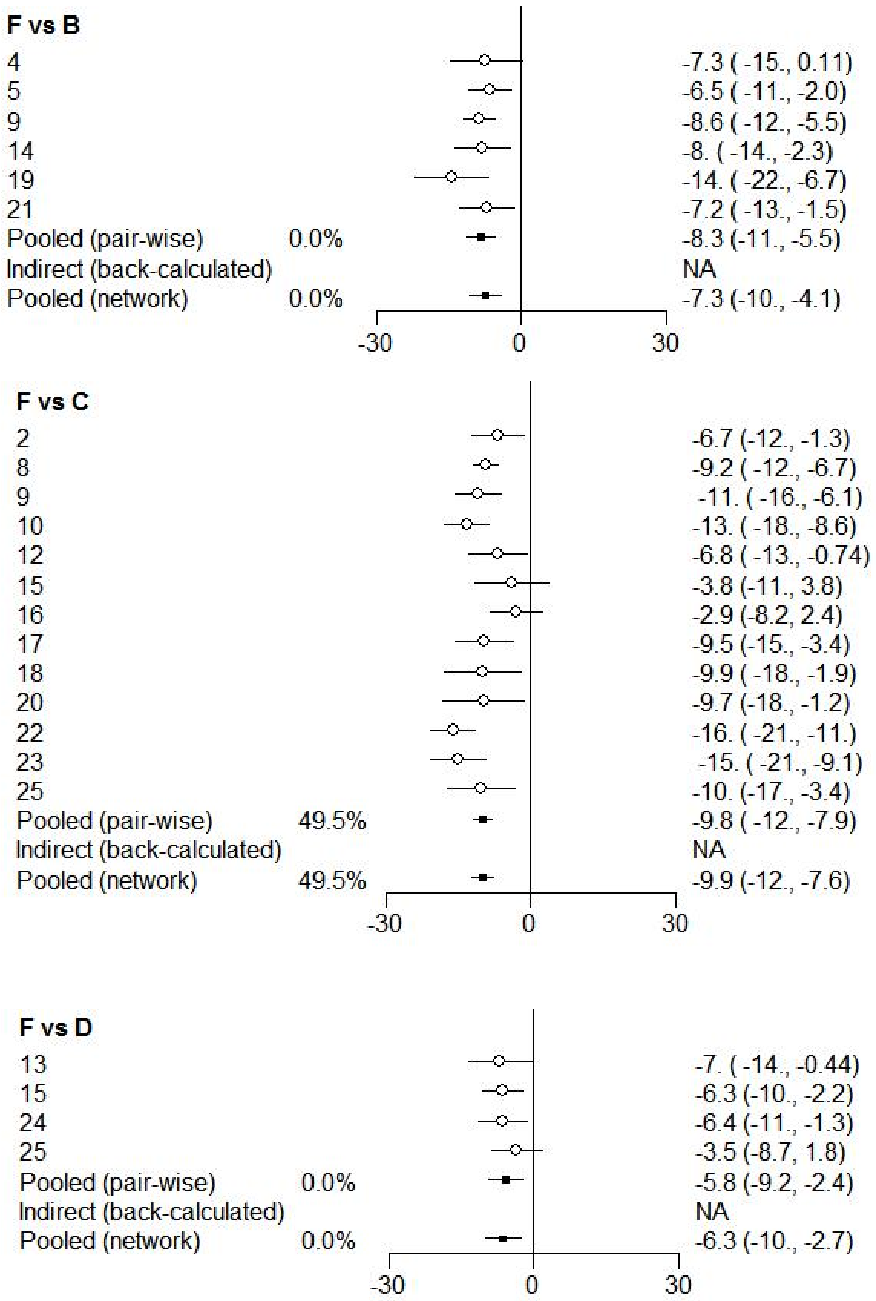

A Low-dose DXM-IV
B High-dose DXM-IV
C DXM-PN
D DEX-PN
E Combined DEX-DXM
F Control groups

```
library(gemtc)
setwd("C:/Users/Administrator/Desktop/x2023")
data1 <- read.csv("f1.csv", sep=",", header=T)
data2 <- read.csv("f2.csv", sep=",", header=T)
head(data1)
head(data2)
devAskNewPage(ask = FALSE)
network <- mtc.network(data1, description = "Example", treatments    = data2)
plot(network)
model <- mtc.model(network, type="consistency", likelihood='normal', link='identity')
results <- mtc.run(model, n.adapt=5000, n.iter = 20000, thin=1)

forest(relative.effect(results,"A"))
plot(results)
gelman.plot(results)
gelman.diag(results)
relative.effect.table(results)
ranks <- rank.probability(results)
print(ranks)
sucra(ranks)
plot(ranks,beside=TRUE)
result.node <- mtc.nodesplit(network, thin=1)
summary.ns <- summary(result.node)
plot(summary.ns)
resultanohe <- mtc.anohe(network, n.adapt=5000, n.iter=20000, thin=1, n.chain=4, likelihood="normal", link="identity", linearModel="random")
c <- summary(resultanohe)
plot(c)
```
