# Supplementary material for: Comparison of dexmedetomidine and dexamethasone as adjuvants to the ultrasound-guided interscalene nerve block in arthroscopic shoulder surgery: a systematic review and Bayesian network meta-analysis of randomized controlled trials
Source: Front Med (Lausanne). 2023 Jun 16;10:1159216. doi: 10.3389/fmed.2023.1159216 (PMC10312098; doi:10.3389/fmed.2023.1159216)
Supplement: Supplementary file 6 [file Data_Sheet_6.pdf]

A Low dose-DXM-IV
B High dose-DXM-IV
C DXM-PN
D DEX-PN
E DEX-DXM
F placebo

Chalifoux et al.
P =0.1574
SUCRA

A 35.6%
B 47.9%
C 77.1%
D 39.4%
E 99.8%
F 0.3%

E	C	B	D	A	
22.25 (16.55,27.95)	9.92 (7.57,12.27)	7.51 (4.10,10.91)	6.79 (3.24,10.34)	6.53 (3.21,9.84)	placebo

Jadon et al.
P =0.1567
SUCRA

A 49.7%
B 55.4%
C 67.5%
D 12.5%
E 99.0%
F 10.9%

E	C	B	A	D	
22.23 (16.64,27.81)	10.01 (7.63,12.38)	7.49 (4.34,10.64)	6.84 (3.35,10.33)	6.74 (3.68,9.80)	placebo

Rodrigues et al.
P =0.9064
SUCRA

A 29.1%
B 54.4%
C 77.8%
D 38.7%
E 99.8%
F 0.3%

E	C	B	D	A	
31.26 (18.60,43.91)	11.33 (1.61,21.05)	8.94 (0.77,18.65)	8.23 (4.62,21.09)	-2.10 (-13.85,9.64)	placebo

Woo et al
P =0.1333
SUCRA

A 36.9%
B 46.8%
C 77.9%
D 38.5%
E 99.5%
F 0.3%

E	C	B	D	A	
7.64 (0.42,19.27)	11.33 (1.48,21.18)	8.93 (0.91,18.77)	9.36 (4.99,25.26)	-2.10 (-14.01,9.80)	placebo

Holland et al
P =0.0651
SUCRA

A 37.5%
B 46.4%
C 76.6%
D 39.4%
E 99.5%
F 0.2%

E	C	B	D	A	
7.64 (0.41,19.17)	7.64 (1.02,17.69)	10.00 (4.88,15.12)	2.36 (0.58,11.16)	0.52 (-11.32,12.36)	placebo

Kang et al.
P =0.2687
SUCRA

A 36.5%
B 49.6%
C 77.8%
D 36.0%
E 99.5%
F 0.2%

E	C	B	A	D	
7.66 (0.23,18.55)	11.36 (2.40,20.31)	8.96 (0.02,17.91)	2.34 (0.52,10.54)	-2.10 (-12.90,8.69)	placebo

Kataria et al
P =0.0925
SUCRA

A 36.8%
B 46.9%
C 77.0%
D 39.3%
E 99.7%
F 0.2%

E	C	B	D	A	
7.64 (0.89,19.16)	11.33 (1.61,21.05)	8.94 (0.77,18.65)	0.36 (0.08,11.16)	-2.10 (-13.85,9.64)	placebo

Chun et al
P =0.0999
SUCRA

A 36.5%
B 49.5%
C 76.2%
D 37.9%
E 99.0%
F 0.3%

E	C	B	D	A	
7.64 (0.89,19.16)	11.33 (1.61,21.05)	8.94 (0.78,18.65)	0.36 (0.08,11.16)	0.24 (-12.19,12.67)	placebo

Desmet et al
P =0.0657
SUCRA

A 37.2%
B 46.4%
C 76.9%
D 39.5%
E 99.0%
F 0.2%

E	C	B	D	A	
7.64 (0.89,19.16)	11.33 (1.61,21.05)	8.94 (0.78,18.65)	0.36 (0.08,11.16)	-2.10 (-13.85,9.64)	placebo

woo et al.
P =0.1397
SUCRA

A	36.7%
B	47.0%
C	77.2%
D	39.1%
E	99.5%
F	0.3%

E	C	B	D	A	
7.63 (0.43,19.30)	11.33 (1.45,21.21)	8.93 (0.94,18.80)	-0.37 (-12.02,11.29)	-2.10 (-14.05,9.84)	placebo

McHardy et al.
P = 0.1133
SUCRA

A	33.7%
B	47.3%
C	78.1%
D	40.9%
E	99.5%
F	0.3%

E	C	B	D	A	
7.64 (0.58,19.16)	11.33 (1.61,21.05)	8.94 (0.77,18.65)	-0.36 (-11.88,11.16)	0.24 (-12.19,12.67)	placebo

Kawanishi et al.
P =0.1276
SUCRA

A	39.6%
B	45.8%
C	77.0%
D	37.5%
E	99.8%
F	0.2%

E	C	B	A	D	
7.63 (0.41,19.36)	11.33 (1.36,21.29)	8.93 (1.02,18.88)	-2.10 (-14.15,9.94)	-0.37 (-12.09,11.36)	placebo

Jung et al.
P =0.1582
SUCRA

A	36.8%
B	46.9%
C	77.0%
D	39.2%
E	99.7%
F	0.3%

E	C	B	D	A	
7.63 (0.42,19.51)	11.32 (1.18,21.46)	8.92 (1.21,19.05)	-0.37 (-12.25,11.50)	-2.10 (-14.37,10.16)	placebo

Lin et al.
P =0.1364
SUCRA

A	40.7%
B	43.7%
C	77.1%
D	38.5%
E	99.8%
F	0.4%

E	C	B	A	D	
9.49 (0.82,21.79)	13.18 (2.54,23.81)	10.78 (0.15,21.41)	-2.10 (-14.25,10.05)	1.49 (-10.82,13.79)	placebo

Margulis et al.
P =0.1265
SUCRA

A	38.9%
B	48.4%
C	78.6%
D	34.1%
E	99.8%
F	0.3%

E	C	B	A	D	
7.64 (0.35,19.04)	11.34 (1.77,20.91)	8.94 (0.62,18.50)	-2.10 (-13.66,9.45)	-0.36 (-11.75,11.03)	placebo

Vasconcelos et al.
P =0.0531
SUCRA

A 36.4%
B 46.8%
C 78.6%
D 38.1%
E 99.7%
F 0.2%

E	C	B	D	A	
7.68 (0.87,18.22)	11.37 (2.84,19.91)	8.98 (0.45,17.50)	0.12 (0.06,10.22)	-2.10 (-12.37,8.17)	placebo

Morita et al.
P =0.1538
SUCRA

A 36.4%
B 46.9%
C 77.4%
D 39.2%
E 99.6%
F 0.1%

E	C	B	D	A	
7.63 (0.21,19.46)	11.32 (1.24,21.40)	8.93 (1.15,19.00)	0.17 (0.20,11.46)	-2.10 (-14.30,10.09)	placebo

Sakae et al.
P =0.2316
SUCRA

A 41.4%
B 46.0%
C 76.4%
D 36.2%
E 99.8%
F 0.2%

E	C	B	A	D	
7.63 (0.41,19.44)	11.32 (1.27,21.38)	8.93 (1.12,18.98)	-2.10 (-14.27,10.06)	-0.37 (-12.18,11.44)	placebo

Yang et al.
P =0.1825
SUCRA

A	36.2%
B	42.1%
C	78.3%
D	43.4%
E	99.7%
F	0.1%

E	C	B	D	A	
5.58 (0.16,17.76)	9.27 (1.22,19.76)	6.87 (0.61,17.35)	-2.42 (-14.60,9.75)	-2.10 (-13.95,9.74)	placebo

JIN et al.
P =0.2612
SUCRA

A	42.6%
B	45.7%
C	75.9%
D	35.8%
E	99.7%
F	0.1%

E	C	B	A	D	
7.63 (0.31,19.56)	11.32 (1.12,21.52)	8.92 (1.27,19.11)	-2.10 (-14.44,10.24)	-0.37 (-12.30,11.55)	placebo

LV et al.
P =0.0530
SUCRA

A	36.3%
B	50.2%
C	77.4%
D	36.4%
E	99.7%
F	0.1%

E	C	B	D	A	
7.66 (0.32,18.55)	11.36 (2.40,20.31)	8.96 (0.02,17.91)	-0.34 (-11.22,10.54)	-2.10 (-12.90,8.69)	placebo

Shen et al.
P =0.0826
SUCRA

A	36.6%
B	47.6%
C	76.8%
D	38.9%
E	99.7%
F	0.2%

E	C	B	D	A	
7.66 (0.30,18.59)	11.36 (2.35,20.36)	8.96 (0.03,17.95)	-0.34 (-11.26,10.58)	-2.10 (-12.96,8.75)	placebo

Yu et al.
P =0.1134
SUCRA

A	50.2%
B	55.2%
C	68.5%
D	11.7%
E	99.0%
F	10.4%

E	C	B	A	D	
7.65 (0.37,18.99)	11.34 (1.84,20.84)	8.94 (0.55,18.43)	-2.10 (-13.58,9.37)	-0.35 (-11.69,10.98)	placebo

Qian et al.
P =0.1583
SUCRA

A	50.0%
B	55.3%
C	67.6%
D	12.1%
E	99.0%
F	10.0%

E	C	B	A	D	
7.63 (0.26,19.51)	11.32 (1.18,21.46)	8.92 (1.21,19.05)	-2.10 (-14.37,10.16)	-0.37 (-12.25,11.50)	placebo

Feng et al.
P =0.0867
SUCRA

A	49.9%
B	55.2%
C	68.5%
D	11.9%
E	98.0%
F	8.5%

E	C	B	A	D	
7.64 (0.37,19.04)	11.34 (1.77,20.91)	8.94 (0.62,18.50)	-2.10 (-13.66,9.45)	-0.36 (-11.75,11.03)	placebo
