# Supplementary figures and images for: Comparison of dexmedetomidine and dexamethasone as adjuvants to the ultrasound-guided interscalene nerve block in arthroscopic shoulder surgery: a systematic review and Bayesian network meta-analysis of randomized controlled trials
Source: Front Med (Lausanne). 2023 Jun 16;10:1159216. doi: 10.3389/fmed.2023.1159216 (PMC10312098; doi:10.3389/fmed.2023.1159216)

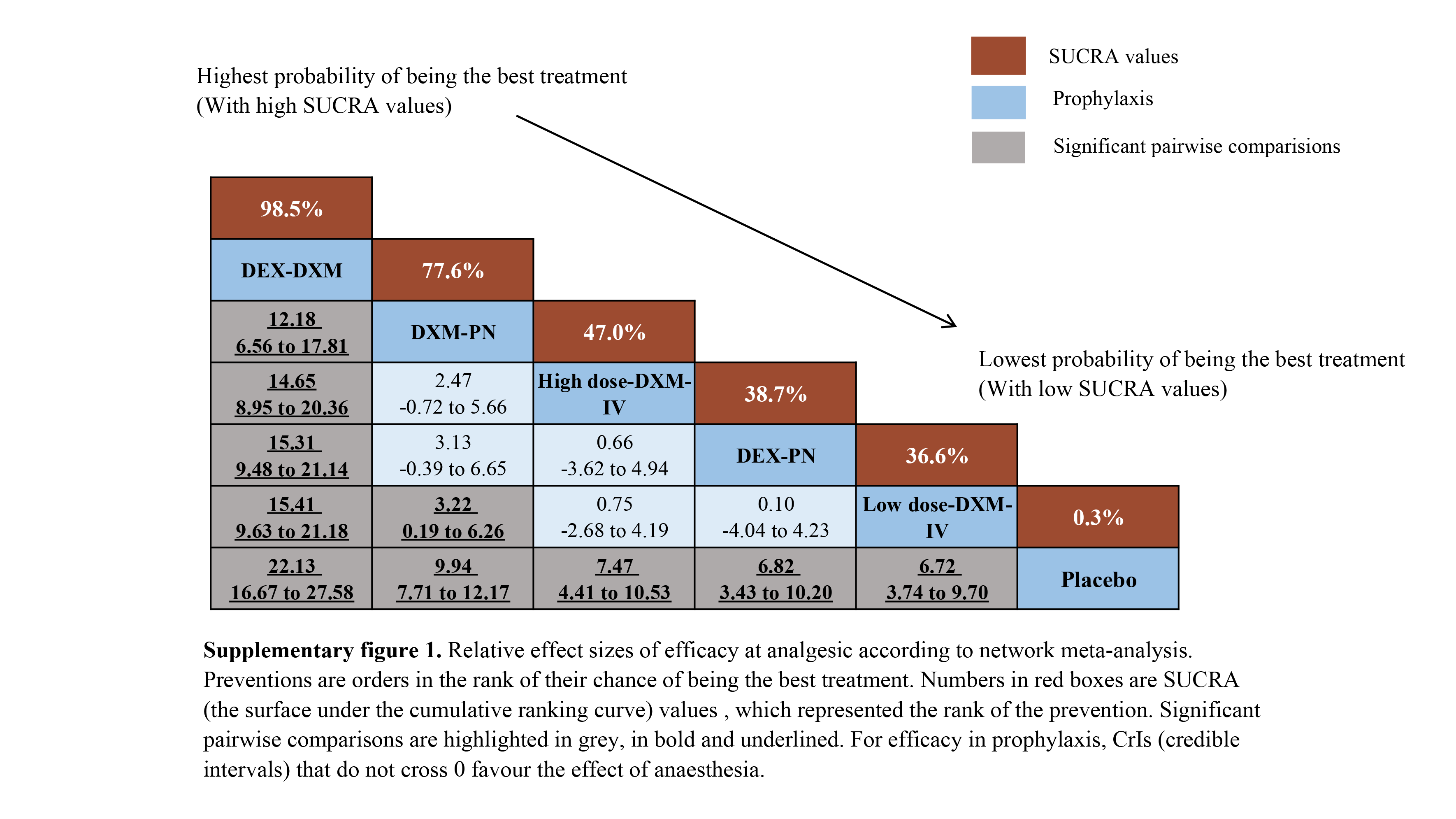

Supplement: Supplementary file 7 [file Image_1.TIF]

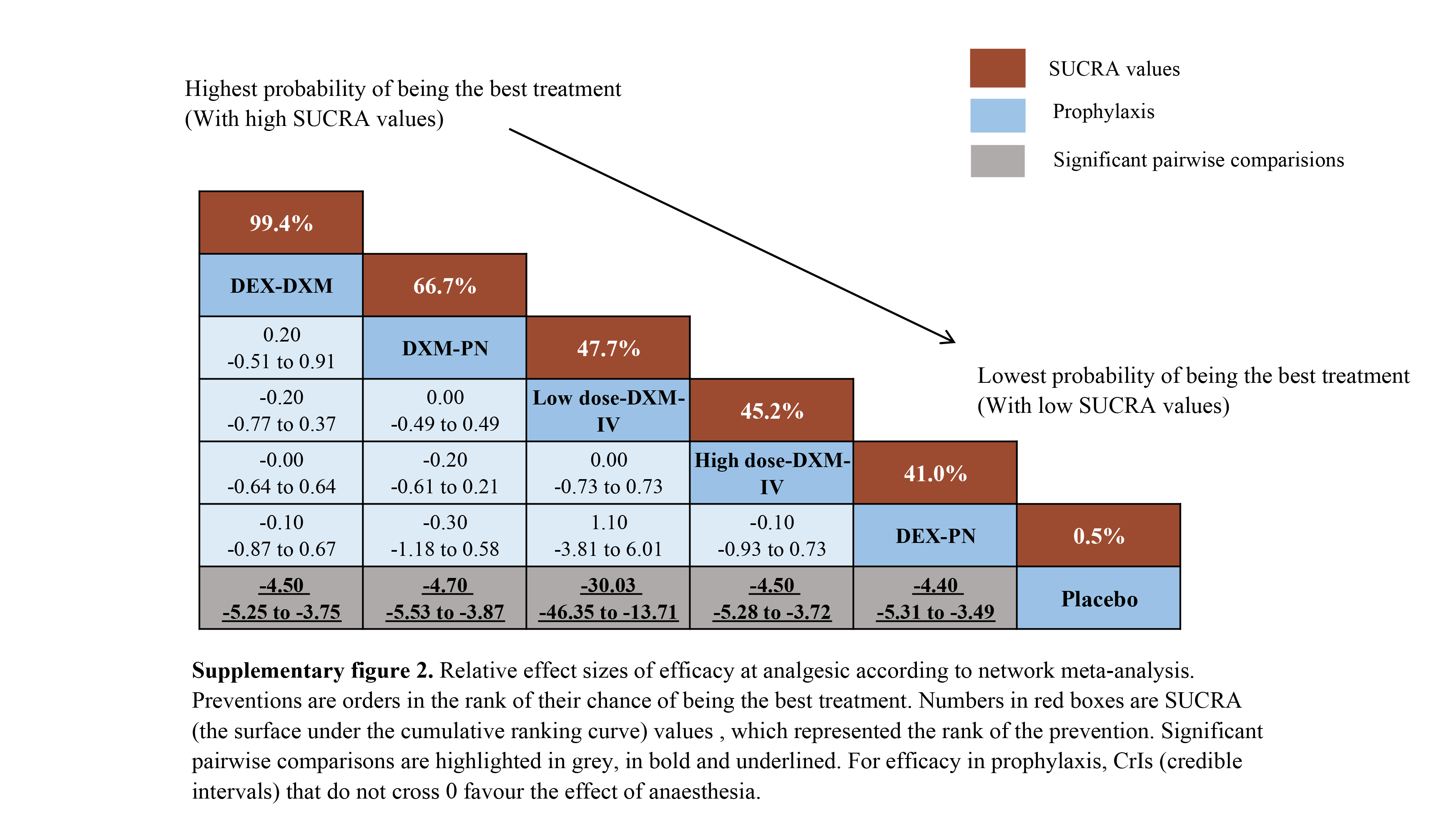

Supplement: Supplementary file 8 [file Image_2.TIF]

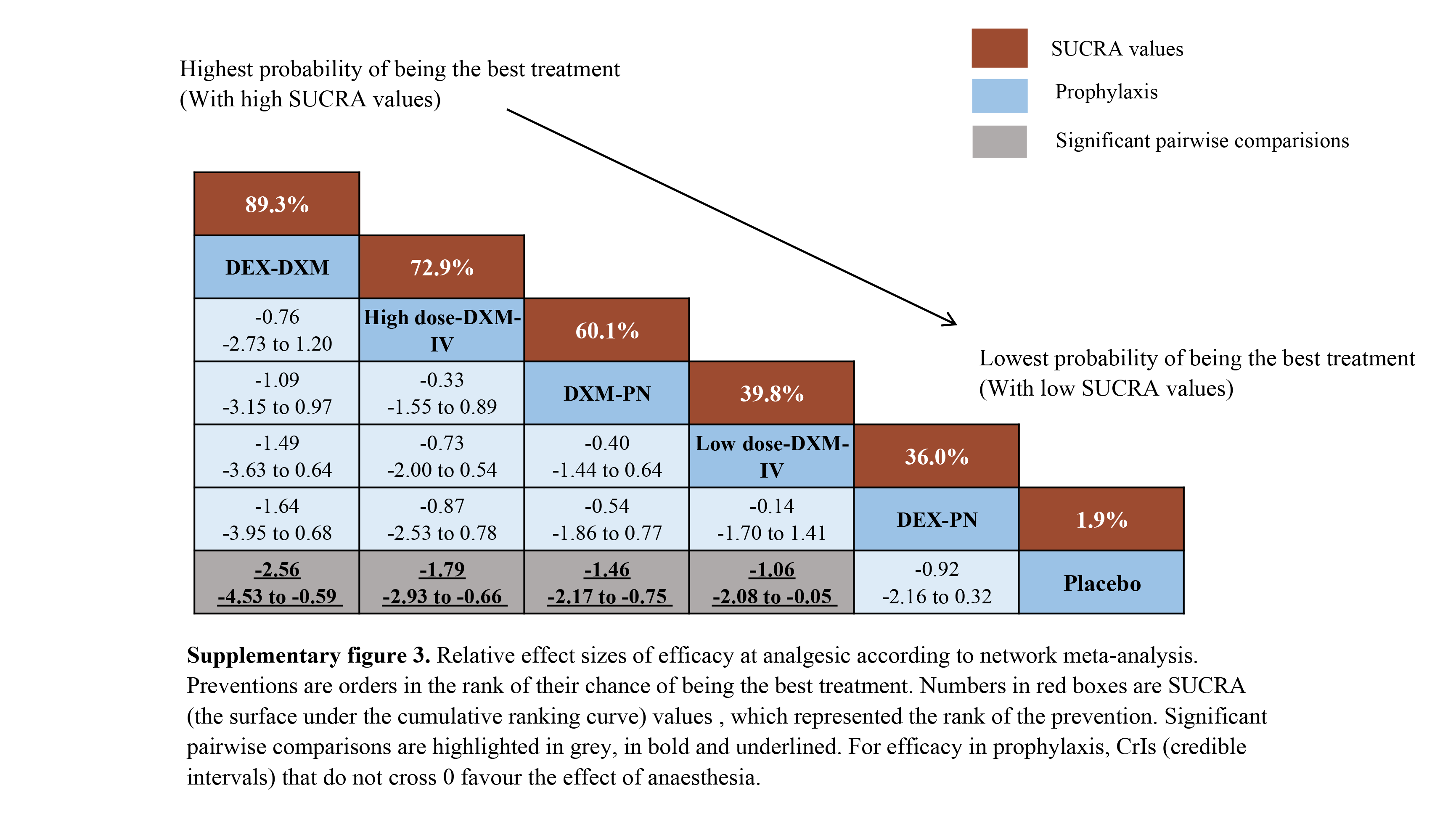

Supplement: Supplementary file 9 [file Image_3.TIF]
